# Assessing the association between household air pollution exposure and child heath in Mongolia: a birth-cohort study

**DOI:** 10.1038/s41598-024-79927-6

**Published:** 2025-01-31

**Authors:** Zaiyou Dai, Katherine E. Woolley, Emma Dickinson-Craig, Tsogzolma Bayandorj, Narangerel Gombojav, Bazarragchaa Tsogt, David Warburton, G. Neil Thomas, Semira Manaseki-Holland

**Affiliations:** 1 Institute of Applied Health Research, University of Birmingham, Birmingham, UK; 2 Public Health Institute Ulaanbaatar, Ulaanbaatar, Mongolia; 3 Child and Adolescent Health Measurement Initiative, Department of Population, Family and Reproductive Heath, Johns Hopkins Bloomberg School of Public Health, Baltimore, MA USA; 4 Mongolian Anti-Tuberculosis Coalition, Research and Innovation Lead, Ulaanbaatar, Mongolia; 5 Children’s Hospital Los Angeles, Saban Research Institute, University of Southern California, Los Angeles, CA USA

**Keywords:** Household air pollution, Mongolia, Child Health, Composite score, BSID-II, Pneumonia, Epidemiology, Environmental impact, Public health

## Abstract

Children in Mongolia are exposed to harmful levels of household air pollution (HAP) due to a high reliance on coal for indoor cooking and heating. This study aims to assess the association between HAP and child health outcomes, in a birth cohort from Ulaanbaatar, Mongolia. A composite HAP measure was created using information on cooking and heating fuels and behaviours collected as part of a randomised control trial assessing the impact of swaddling on child health. Child health outcomes (Bayley Scales of Infant Development scores [BSID-II], pneumonia, height and weight) were collected at 7, 13, and 36 months. Linear and Cox proportional hazard model were used to assess the association between HAP and child health outcomes at each time point, adjusting for child, maternal and environmental confounding factors. An increased risk of pneumonia was observed with an increasing HAP score (adjusted hazard ratio: 1.02 [1.01, 1.04]) at 7 months). An increase in HAP exposure was associated with a decrease in the BSID mental score at 13 months (β: − 0.09 [− 0.17, − 0.01]), BSID psychomotor score at 36 months (β: − 0.12 [− 0.23, − 0.02]). A decrease in height-for-age z-score (HAZ) and weight-for-age z-score (WAZ) were associated with increased HAP exposure at 7 (HAZ β: − 0.019 [− 0.030, − 0.010] and 13 months (HAZ β: − 0.020 [− 0.030, − 0.011], and WAZ β: − 0.012 [− 0.019, − 0.005]), however only HAZ was associated with HAP at 36 months (β: − 0.011 [− 0.020, − 0.002]). An increasing HAP score was associated with an increase in the health outcome composite score at 7 months only (β: 0.019; 95% CI 0.003–0.035). HAP exposure was shown to negatively impact child health sustainably over 3 years. There are implications for development of appropriate public health policies to mitigate HAP exposure throughout Mongolia and similar Central Asia settings.

**Supplementary Information:**

The online version contains supplementary material available at 10.1038/s41598-024-79927-6.

## Introduction

Household air pollution (HAP) from cooking, heating and lighting using solid biomass fuel (wood, dung, charcoal, and crop residue) and coal, causes significant morbidity and mortality and is a global public health concern in low- and middle-income countries (LIMCs)^1^. Evidence on the impact of air pollution on health is still expanding. Based on the current evidence, globally there are 3.8 million premature deaths^2^ and 110 million disability-adjusted life years attributable to HAP each year^3^, with health events associated with HAP occurring through the life course^4^. Women and children are particularly vulnerable to HAP due to often spending a greater amount of time within the home than other household members^5^. In addition, the immature physiology of children increases their vulnerability to HAP^6^. Health events associated with HAP occur with pregnancy (e.g., low-birthweight, pre-term birth, preeclampsia)^7^ and continue into childhood (e.g., acute respiratory infection (ARI), asthma, burns, neurodevelopmental disorders, stunting)^8,9^ and adulthood, (e.g., asthma, chronic obstructive pulmonary disease [COPD], cardiovascular disease, cognition)^2,10–12^. However, studies that investigate the health implications of HAP exposure are often cross-sectional or experimental in nature^13^ and lack longitudinally^8^. Few studies use birth cohorts to investigate child health impacts from HAP in LIMCs, but those that have done so only investigated health events in the first^14^, second^15^ and fourth year of life^16^. In addition, there is a paucity of evidence in the literature around the use of composite health outcome measures, for multiple health outcomes. There are also environmental (e.g., landslide, erosion etc.)^17,18^ and socioeconomic (e.g., opportunity costs, gender inequality, healthcare cost)^5,19–22^ implications associated with the reliance and exposure to HAP producing fuels.

Mongolia is a land-locked lower-middle-income country in north-central Asia, positioned on a plateau surrounded by high mountain ranges. The capital city of Mongolia, Ulaanbaatar, holds over half the population (1,900,000 people 2023) and often reaches temperatures of − 20 °C, resulting in heavy reliance on household heating to stay warm^23^. Mongolia has four seasons, winter (November-February), spring (March-May), summer (June-August) and autumn (September-October). Sixty-percent of the capital’s population live in the so-called ger districts surrounding the city, which comprise of both traditional Mongolian felt tents called gers and rudimentary wooden/brick houses. The vast majority of such households have a reliance on coal fired stoves for cooking and heating, burning approximately 3–6 tons of coal each winter, resulting in hazardous levels of indoor and outdoor air pollution^24,25^. The remaining 40% of the population live mainly in apartments, which have central heating^23^. In other parts of the country, as with many other surrounding Central Asian regions^26^, similar ger structures or wooden housing structures also have similar stoves and burn coal or wood with high indoor HAP exposure for the young in winters. Despite HAP being a health threat, the majority of research into air pollution in Ullaanbaatar focuses on ambient air pollution, as it is deemed to be the greater problem.

Characterising and documenting an accurate level of exposure is a challenge for epidemiological studies. Self-reported exposures or measurement of particulate matter (PM) and carbon monoxide (CO) are commonly collected to estimate HAP exposure. Direct methods of collection are time consuming and expensive, often limiting the sample size^27,28^. Fuel type, stove type, and cooking location have previously been used to assess relative differences in HAP exposure, providing large-scale results, but are limited by the availability of information and not all factors that affect HAP concentrations can be taken into account. The use of evidence-based composite measures of HAP exposures allows for multiple factors that influence HAP concentrations (e.g., ventilation, fuel type, stove type) to be taken into consideration, giving a potential more accurate estimation of HAP exposure. However, there is a paucity of evidence for the use of composite HAP measures to understand the associated health impacts.

Therefore, this study aims to address the gap in the literature through multiple objectives: (a) demonstrate the feasibility of the use of a composite survey based air pollution score to assess exposure (b) assess over the first three years of life, the score and association between HAP and (i) child development using the Bayley Test, (ii) hospital-confirmed pneumonia, (iii) stunting (height-for-age z-score [HAZ]) and being underweight (weight-for-age z-score [WAZ]), as well as a (iv) health outcome composite score, in a birth cohort from Ulaanbaatar, Mongolia.

## Results

### Baseline characteristics

A HAP score could be calculated for 1266 (99%) children, with no observed difference in baseline characteristics with those children without a score (*n* = 13; 1%). Of the included children 421 (33.3%) resided in the lowest polluted households, 293 (23.1%) in low polluted households, 217 (17.1%) in medium polluted household, and 335 (26.5%) in highly polluted households. There was an even split between males (50.4%) and females (49.1%) (Table 1). Mean mental and psychomotor BSID scores were 105.5 (10.6) and 100.0 (16.0) for children at 13-months and 92.4 (11.0) and 100.9 (11.3) for children at 36-months. Integrated management of childhood illness (IMCI) pneumonia was identified in 24.8% of infants in the 7-months following birth (Table 1). HAZ scores at 7-, 13-, and 36-months had median values of 0.27 (IQR: − 0.44–1.19), − 0.61 (IQR: − 1.29–0.16) and − 0.85 (IQR: − 1.52 – − 0.16) respectively. Whereas WAZ scores at 7-, 36-, and 36-months had median values of 0.62 (IQR: − 0.06–1.28), 0.22 (IQR: − 0.37–0.83), and 0.08 (IQR: − 0.46–0.56), respectively. The composite health outcome score was found to have a median score of 7 (IQR: 5–8) at 7-months, 10 (IQR: 8–12) at 13-months, and 10 (IQR: 9–12) at 36-months (Supplementary Appendix 1).

**Table 1:** Baseline characteristics categorised by composite household air pollution score.

Variables	Composite household air pollution score				
	Total (*n* = 1266)	Lowest (*n* = 421)	Low (*n* = 293)	Medium (*n* = 217)	High (*n* = 335)
Swaddling, n (%)					
Yes	635 (50.2)	201 (47.7)	142 (48.5)	117 (53.9)	175 (52.2)
No	631 (49.8)	220 (52.3)	151 (51.5)	100 (46.1)	160 (47.8)
Sex, n (%)					
Female	622 (49.1)	203 (48.2)	147 (50.2)	105 (48.4)	167 (49.9)
Male	638 (50.4)	216 (51.3)	145 (49.5)	111 (51.2)	166 (49.6)
Missing value	6 (0.5)	2 (0.5)	1 (0.3)	1 (0.5)	2 (0.6)
Birthweight, n (%)					
2400–2950 g	142(11.2)	29 (6.9)	42 (14.3)	30 (13.8)	41 (12.2)
3000–3450 g	543 (42.9)	192 (45.6)	119 (40.6)	96 (44.2)	136 (40.6)
3500–3950 g	425 (33.6)	144 (34.2)	103 (35.2)	63 (29.0)	115 (34.3)
≥ 4000 g	145 (11.5)	54 (12.8)	26 (8.9)	26 (12.0)	39 (11.6)
Missing value	11 (0.9)	2 (0.5)	3 (1.0)	2 (0.9)	4 (1.2)
Gestational age, n (%)					
36–37 weeks	41 (3.2)	15 (3.6)	12 (4.1)	5 (2.3)	29 (2.7)
≥ 38 weeks	1167 (92.2)	397 (94.3)	263 (89.8)	200 (92.2)	307 (91.6)
Missing value	58 (4.6)	9 (2.1)	18 (6.1)	12 (5.5)	19 (5.7)
Type of delivery, n (%)					
Vaginal	1051 (83.0)	331 (78.6)	251 (85.7)	179 (82.5)	290 (86.6)
Caesarean	203 (16.0)	86 (20.4)	40 (13.7)	35 (16.1)	42 (12.5)
Missing value	12 (1.0)	4 (1.0)	2 (0.7)	3 (1.4)	3 (0.9)
Parity, n (%)					
0	581 (45.9)	225 (53.4)	131 (44.7)	92 (42.4)	133 (39.7)
1 or 2	202 (16.0)	62 (14.7)	48 (16.4)	37 (17.1)	55 (16.4)
3	327 (25.8)	89 (21.1)	81 (27.6)	57 (26.3)	100 (29.9)
Missing value	156 (12.3)	49 (10.7)	33 (11.26)	31 (14.3)	47 (14.0)
Breastfeeding until 4-months, n (%)					
Not breastfed	99 (7.8)	40 (9.5)	15 (5.1)	18 (8.3)	26 (7.8)
Nonexclusively breastfed	1137 (89.8)	369 (87.6)	272 (92.8)	194 (89.4)	302 (90.1)
Exclusively breastfed	30 (2.4)	12 (2.9)	6 (2.0)	5 (2.3)	7 (2.1)
Maternal marital status, n (%)					
Common law	574 (45.3)	196 (46.6)	147 (50.2)	95 (43.8)	136 (40.6)
Married	655 (51.7)	220 (52.3)	135 (46.1)	114 (52.5)	186 (55.5)
Single	33 (2.6)	5 (1.2)	10 (3.4)	7 (3.2)	11 (3.3)
Missing value	4 (0.3)	0 (0.0)	1 (0.3)	1 (0.5)	2 (0.6)
Maternal age, n (%)					
< 20 years	95 (7.5)	23 (5.7)	24 (8.2)	20 (9.2)	28 (8.4)
20–29 years	847 (66.9)	275 (65.3)	199 (67.9)	144 (66.4)	229 (68.4)
≥ 35 years	319 (25.2)	122 (29.0)	69 (23.6)	52 (24.0)	76 (22.7)
Missing value	5 (0.4)	1 (0.2)	1 (0.3)	1 (0.5)	2 (0.6)
Maternal education, n (%)					
Primary school	143 (11.3)	18 (4.3)	40 (13.7)	32 (14.7)	53 (15.8)
Secondary school	682 (53.9)	150 (35.6)	186 (63.5)	130 (59.9)	216 (64.5)
Tertiary school	438 (34.6)	253 (60.1)	66 (22.5)	54 (24.9)	65 (19.4)
Missing value	3 (0.2)	0 (0.0)	1 (0.3)	1 (0.5)	1 (0.3)
Mothers’ employment, n (%)					
No	213 (16.8)	73 (17.3)	47 (16.0)	33 (15.2)	60 (17.9)
Yes	986 (77.9)	329 (78.1)	230 (78.5)	172 (79.3)	255 (76.1)
Missing value	67 (5.3)	19 (4.5)	16 (5.5)	12 (5.5)	20 (6.0)
Post-natal depression, n (%)					
Normal	1025 (81.0)	350 (83.1)	237 (80.9)	167 (77.0)	271 (80.9)
Depressed	168 (13.3)	50 (11.9)	42 (14.3)	30 (13.8)	46 (13.7)
Missing value	73 (5.8)	21 (5.0)	14 (4.8)	20 (9.2)	18 (5.4)
Number of smokers in household, n (%)					
None	607 (47.9)	224 (53.2)	142 (48.5)	91 (41.9)	150 (44.8)
One smoker	544 (43.0)	162 (38.5)	130 (44.4)	100 (46.1)	152 (45.4)
More than one smoker	115 (9.1)	35 (8.3)	21 (7.2)	26 (12.0)	33 (9.9)

Proportion of missing data for total sample: sex = 0.5%, type of delivery = 0.9%, parity = 12.3%, maternal marital status = 0.3%, maternal education = 5.3%, depression = 5.8%.

*n* number of observations, % percentage, *missing data present.

### BSID-II

Both mental and psychomotor BSID scores were associated with HAP exposure at 13- and 36-months in the unadjusted analysis (Table 2). After adjusting for child, maternal, and environmental confounding factors, being exposed to higher levels of HAP was associated with lower mental BSID score at 13-months (β: − 0.09; 95% CI − 0.17, − 0.01) and lower psychomotor scores at 36-months (β: − 0.12; 95% CI − 0.23, − 0.02). The same decrease with BSID score for psychomotor at 13-months and mental at 36-months was observed but was not statically significant.

**Table 2:** Multiple linear regression of the association between HAP scores and BSID scores.

	13-months				36-months			
	Mental		Psychomotor		Mental		Psychomotor	
	Mean (SD)	β (95% CI)	Mean (SD)	β (95% CI)	Mean (SD)	β (95% CI)	Mean (SD)	β (95% CI)
Unadjusted	*N* = 1088	− 0.13 (− 0.19, − 0.06)	*N* = 1094	− 0.15 (− 0.25, − 0.05)	*N* = 685	− 0.24 (− 0.32, − 0.15)	*N* = 687	− 0.25 (− 0.33, − 0.16)
	105.50 (10.63)	*P* < 0.001	99.96 (15.96)	*P* = 0.003	92.43 (11.01)	*P* < 0.001	100.90 (11.27)	*P* < 0.001
Model 1	*N* = 1038	− 0.10 (− 0.17, − 0.04)	*N* = 1044	− 0.13 (− 0.22, − 0.02)	*N* = 649	− 0.23 (− 0.31, − 0.14)	*N* = 652	− 0.24 (− 0.33, − 0.15)
	105.48 (10.69)	*P* = 0.002	100.01 (15.89)	*P* = 0.021	92.50 (11.00)	*P* < 0.001	100.99 (11.28)	*P* < 0.001
Model 2	*N* = 855	− 0.08 (− 0.16, − 0.00)	*N* = 862	− 0.08 (− 0.20, 0.04)	*N* = 550	− 0.08 (− 0.18, 0.02)	*N* = 552	− 0.13 (− 0.23, − 0.30)
	105.23 (10.66)	*P* = 0.049	99.73 (15.95)	*P* = 0.217	92.61 (11.13)	*P* = 0.115	101.12 (11.06)	*P* = 0.011
Model 3	*N* = 851	− 0.09 (− 0.17, − 0.01)	*N* = 858	− 0.09 (− 0.21, 0.03)	*N* = 546	− 0.09 (− 0.19, 0.01)	*N* = 548	− 0.12 (− 0.23, − 0.02)
	105.26 (10.65)	*P* = 0.035	99.78 (15.97)	*P* = 0.154	92.53 (11.11)	*P* = 0.092	101.06 (11.04)	*P* = 0.016

Model 1: Adjusted for sex, birthweight, gestational age, date of birth.

Model 2: Model 1 plus parity, maternal marital status, maternal age, maternal education, maternal employment, postnatal depression.

Model 3: Model 2 plus type of delivery, swaddling, breastfeeding, number of smokers.

Associated covariates can be found in Supplementary Appendix 1.

*β* Coefficient, *95% CI* 95% confidence intervals, *n* number of observations within the model, *mean* mean score of observation included within the model, *SD* standard deviation of the mean.

### Pneumonia

When assessing the HAP score as a continuous variable, the hazard ratio for pneumonia increased by 2% (95% CI: 1, 4) for every increase in the HAP score (Table 3). This increase in the hazards ratio with higher HAP scores was also observed when assessing the HAP scores as a categorical variable. Low HAP score had a 52% (4-124) increase, medium HAP scores a 71% (15–154) increase and high HAP score of an 80% (25–159) increase in the hazards ratio of pneumonia compared to the lowest HAP score; with the hazard ratio increasing in size from low to high HAP scores.

**Table 3:** Survival analysis of the association between HAP and pneumonia.

HAP	Number of infants	Total number of child days	Total cases	Unadjusted	Adjusted		
				(HR (95% CI))	(AHR (95% CI))		
				*n* = 1190	Model 1 *n* = 1132	Model 2 *n* = 934	Model 3 *n* = 930
HAP score	1190	219,699	315	1.03 (1.02, 1.04)	1.03(1.01, 1.04)	1.02 (1.01, 1.04)	1.02 (1.01, 1.04)
				*P* < 0.001	*P* < 0.001	*P* = 0.004	*P* = 0.002
Lowest	394	75,766	73	1	1	1	1
Low	273	49,875	72	1.50 (1.08, 2.08)	1.43 (1.02, 2.00)	1.42 (0.97, 2.08)	1.52 (1.04, 2.24)
				*P* = 0.014	*P* = 0.036	*P* = 0.073	*P* = 0.032
Medium	206	37,295	65	1.81 (1.30, 2.53)	1.81 (1.29, 2.56)	1.67 (1.13, 2.48)	1.71 (1.15, 2.54)
				*P* < 0.001	*P* = 0.001	*P* = 0.011	*P* = 0.008
High	317	56,763	105	1.94 (1.44, 2.61)	1.91 (1.40, 2.60)	1.75 (1.21, 2.52)	1.80 (1.25, 2.59)
				*P* < 0.001	*P* < 0.001	*P* = 0.002	*P* = 0.002

Model 1: Adjusted for sex, birthweight, gestational age, date of birth.

Model 2: Model 1 plus parity, maternal marital status, maternal age, maternal education, maternal employment, postnatal depression.

Model 3: Model 2 plus type of delivery, swaddling, breastfeeding, number of smokers.

Associated covariates can be found in Supplementary Appendix 1.

*β* Coefficient, *95% CI* 95% confidence intervals, *n* number of observations within the model.

### Height for age (HAZ) and weight for age (WAZ)

In the adjusted analysis HAZ and WAZ were associated with the HAP score at all three timepoints (Table 4). After adjusting for confounders only HAZ at 7-months was observed to be associated with every increase in the HAP score lowering the HAZ score by − 0.019 (95% CI: − 0.030, − 0.010). The same effect was also observed at 13-months, after adjusting for confounding. At 36-months, only HAZ was associated with the HAP score, with a − 0.011 (95% CI: − 0.020, − 0.002) decreased in the HAZ score with every increase in the HAP score.

**Table 4:** Multiple linear regression of the association between HAP scores with height for age z score (HAZ) and weight for age z-score (WAZ) at 7-, 13-, and 36-months.

	7-months				13-months				36-months			
	HAZ		WAZ		HAZ		WAZ		HAZ		WAZ	
	Mean (SD)	β (95% CI)	Mean (SD)	β (95% CI)	Mean (SD)	β (95% CI)	Mean (SD)	β (95% CI)	Mean (SD)	β (95% CI)	Mean (SD)	β (95% CI)
Unadjusted	*N* = 889	− 0.031 (− 0.039, − 0.022)	*N* = 890	− 0.017 (− 0.025, − 0.011)	*N* = 999	− 0.026 (− 0.034, − 0.019)	*N* = 1000	− 0.014 (− 0.021, − 0.008)	*N* = 713	− 0.022 (− 0.030, − 0.014)	*N* = 713	− 0.013 (− 0.019, − 0.006)
	0.376 (1.262)	*P* < 0.001	0.629 (1.041)	*P* < 0.001	− 0. 602 (1.157)	*P* < 0.001	0.247 (0.959)	*P* < 0.001	− 0.865 (1.103)	*P* < 0.001	0.069 (0.820)	*P* < 0.001
Model 1	*N* = 850	− 0.029 (− 0.037, − 0.020)	*N* = 851	− 0.014 (− 0.021, − 0.007)	*N* = 951	− 0.026 (− 0.033, − 0.018)	*N* = 952	− 0.013 (− 0.019, − 0.007)	*N* = 676	− 0.021 (− 0.028, − 0.013)	*N* = 676	− 0.011 (− 0.017, − 0.005)
	0.379 (1.267)	*P* < 0.001	0.637 (1.046)	*P* < 0.001	− 0.599 (1.169)	*P* < 0.001	0.252 (0.968)	*P* < 0.001	− 0.865 (1.045)	*P* < 0.001	0.074 (0.829)	*P* < 0.001
Model 2	*N* = 704	− 0.021 (− 0.030, − 0.011)	*N* = 704	− 0.008 (− 0.017, 0.000)	*N* = 786	− 0.021 (− 0.030, − 0.012)	*N* = 787	− 0.012 (− 0.019, − 0.005)	*N* = 572	− 0.011 (− 0.020, − 0.002)	*N* = 572	− 0.007 (− 0.014, 0.000)
	0.385 (1.273)	*P* < 0.001	0.674 (1.045)	*P* = 0.040	− 0.582 (1.195)	*P* < 0.001	0.285 (0.971)	*P* = 0.002	− 0.864 (1.042)	*P* = 0.015	0.090 (0.828)	*P* = 0.043
Model 3	*N* = 700	− 0.019 (− 0.030, − 0.010)	*N* = 700	− 0.008 (− 0.016, 0.000)	*N* = 782	− 0.020 (− 0.030, − 0.011)	*N* = 783	− 0.012 (− 0.019, − 0.005)	*N* = 568	− 0.011 (− 0.020, − 0.002)	*N* = 568	− 0.007 (− 0.014, 0.000)
	0.380 (1.271)	*P* < 0.001	0.672 (1.046)	*P* = 0.059	− 0.586 (1.195)	*P* < 0.001	0.284 (0.972)	*P* = 0.002	− 0.869 (1.040)	*P* = 0.021	0.088 (0.829)	*P* = 0.052

Model 1: Adjusted for sex, birthweight, gestational age, date of birth.

Model 2: Model 1 plus parity, maternal marital status, maternal age, maternal education, maternal employment, postnatal depression.

Model 3: Model 2 plus type of delivery, swaddling, breastfeeding, number of smokers.

Associated covariates can be found in Supplementary Appendix 1.

*β* Coefficient, *95% CI* 95% confidence intervals, *n* number of observations within the model.

### Composite health measure

At 7-months there was an observed association in the adjusted analysis with every increase in HAP score, increasing the combined health score (pneumonia, stunting, underweight) by 0.020 (95% CI 0.004–0.036) (Table 5). However, no association was observed between the HAP score and combined health score (BSID-II, stunting, underweight) at 13 (β: 0.021; 95% CI − 0.001, 0.042) or 36-months (β: − 0.001; 95% CI − 0.024, 0.023). A further analysis adjusting for a change of residence within the 36-month follow-up period had little impact on the association between HAP score and combined health score at 36-months (Supplementary Appendix 2).

**Table 5:** Multiple linear regression of the association between HAP and composite health score at 7-, 13-, and 36-months.

	7-months		13-months		36-months	
	Mean (SD)	β (95% CI)	Mean (SD)	β (95% CI)	Mean (SD)	β (95% CI)
Unadjusted	*N* = 846	0.033 (0.019, 0.047)	*N* = 979	0.024 (0.006, 0.041)	*N* = 673	− 0.008 (− 0.027, 0.012)
	6.614 (1.996)	*P* < 0.001	10.066 (2.701)	*P* ≤ 0.001	10.281 (2.457)	*P* = 0.451
Model 1	*N* = 828	0.028 (0.015, 0.042)	*N* = 932	0.026 (0.007, 0.044)	*N* = 638	− 0.008 (− 0.028, 0.011)
	6.589 (2.001)	*P* < 0.001	10.065 (2.72)	*P* = 0.005	10.290 (2.447)	*P* = 0.414
Model 2	*N* = 685	0.021 (0.005, 0.037)	*N* = 769	0.023 (0.002, 0.045)	*N* = 539	0.000 (− 0.023, 0.023)
	6.521 (1.987)	*P* = 0.009	9.951 (2.713)	*P* = 0.033	10.24 (2.483)	*P* = 0.996
Model 3	*N* = 681	0.020 (0.004, 0.036)	*N* = 764	0.021 (− 0.001, 0.042)	*N* = 535	− 0.001 (− 0.024, 0.023)
	6.529 (1.987)	*P* = 0.013	9.963 (2.706)	*P* = 0.056	10.237 (2.483)	*P* = 0.955

Model 1: Adjusted for sex, birthweight, gestational age, date of birth.

Model 2: Model 1 plus parity, maternal marital status, maternal age, maternal education, maternal employment, postnatal depression.

Model 3: Model 2 plus type of delivery, swaddling, breastfeeding, number of smokers.

Associated covariates can be found in Supplementary Appendix 1.

*β* Coefficient, *95% CI* 95% confidence intervals, *n* number of observations within the model.

## Discussion

This novel study investigating the effect of HAP exposure on child health, using a Mongolian birth cohort, with a large follow-up period (36-months), utilises a unique approach to estimating the level of HAP exposure through a composite score of air pollution sources and behavioural factors. The results indicate that exposure to HAP negatively impact child health within this setting sustainably over a 3-year period. Therefore, there is evident need for interventions to reduce the level of HAP in Mongolia and similar settings across Central Asia, to improve child health, which will have implications for the sustainable development goal 3 (Good health and well-being). This evidence endorses the findings of other studies that multiple health outcomes are affected by air pollution, which we demonstrate in one birth cohort for the impact on development, pneumonia (the leading cause of death in LMIC young children), and growth (indicator for other health outcomes).

The impacts of HAP on child health are well documented within the literature^29^, including pneumonia^8,30,31^, stunting^32^, underweight^33^, and, child growth^14^; which correspond with the results of this study. However, the existing research on the negative effects of HAP exposure on child neurodevelopment is more limited^34,35^ to which our study contributes significantly by demonstrating a sustained effect using a highly sensitive BSII scores across time from 13-months to 36-months. This negative effect is also seen in children exposed to high levels of ambient air pollution who have lower child development scores^36^. In Ulaanbaatar specifically, a recent RCT identified a positive association between reduction in HAP exposure during pregnancy and child cognitive development^37^. The results of the present study are robustly adjusted for and have statistical significance, but it is not clear what the clinical significance would be, with the BSID score, HAZ, and WAZ, as the relatively low associated average reduction in the score at individual level may not change the overall health status of the individual child (e.g., cross over a threshold). On a population level however, even small improvements in the outcome could result in a shift in the curve, which may well be clinically significant for the population at large. There is a known biological mechanism for the effect on child health, with CO and PM, from both exposure during pregnancy and after birth^7,29^, along with critical exposure time point being identified for different health outcomes^38–41^. However, this study does not explore the difference or change of risk with health outcomes from 12 to 36 months and further research is required to understand the critical points of exposure on determining the occurrence of developmental changes or growth of children. There is evidence of pre-natal and early life exposure resulting in respiratory health consequences, within the first seven months, suggesting a high-risk period. Developmental effects persist across 13 and 36 months, however, at 36 months motor development can be seen to be more prominent, which could be explained by motor skills being likely to be more limited at 13 months. Furthermore, WAZ and HAZ also have sustained long term effects. Although further research is required to identify critical times windows for exposure and expression of health events, this study does indicate that HAP effects on child health is prominent from a young age, suggesting that this is a particularly vulnerable period for exposure to HAP.

It is significant to note that our study was conducted at a time when the ambient (outside) air pollution in Ulaanbaatar was not as high as it is now and there was less awareness of the health threats in Mongolia^42^. Over the past decade, increased rural to urban migration and rapid modern urbanisation have increased the capital’s resident’s exposure to air pollution, through emissions from low-income housing, motor vehicles and power plants^25^. Subsequently, children in Mongolia today are exposed to both indoor household and ambient air pollution indicating that the risk to children in Ulaanbaatar in recent years is many folds higher and likely has a much higher rate of effect than the rates and scores in this study^37^. Whilst as a result of a steady increase of the population’s air pollution exposure, there have been multiple local air pollution mitigation strategies implemented over the past decade, including the recent 2019 ban on raw coal, Ulaanbaatar’s residents continue to be exposed to both hazardous indoor and outdoor pollution levels^43,44^. Our findings are deemed to be invaluable for assessing temporal changes in air pollution exposure and its related impact on health in Ulaanbaatar, which in turn could improve our understanding on the effectiveness of such mitigation strategies. The focus in Mongolia, however, has not been on reducing HAP in household living outside of the city of Ulaanbaatar, while our study demonstrates that these millions of children in Mongolia living outside of Ulaanbaatar and in neighbouring Central Asia^26^ where housing resembles the situation in Mongolia remain at high risk of adverse health outcomes. Our results are relevant to all children and pregnant women exposed to air pollution due to the collection of biological data and use of robust methods for health outcomes measures (e.g., BSID-II). However, extended longitudinal (beyond 3 years) and life-course approaches need to be undertaken within these settings to understand the long-term heath effect and changing health risks.

Another novel aspect of our study is in the methodological assessment of a new low-cost method of measuring HAP. The use of a composite measure of estimated HAP exposure, is an innovative technique and has enabled the quantification of multiple components that affect the HAP concentrations within a household. It is important to demonstrate evidence of validity of such a new method of HAP exposure estimation. We have achieved this through using published evidence^45–47^ on the importance of each component of the score and its effect on HAP, taking into consideration both sources of HAP (e.g., heating source, types of fuels, fuel condition^48,49^) and behaviours (e.g., stove use, fuel handling practices, opening of windows^13,48,50,51^) which influence HAP concentrations. We demonstrated a gradient between high and low exposure that can be seen within these analyses supporting the validity of the composite measure and the existing evidence that the continued reduction in HAP can lead to the potential for benefit with behavioural change (e.g., use of dry fuel, opening doors and windows etc.) in the short-term^52^. Although the use of a composite HAP score requires further validation, including comparisons to measured levels of air pollutants (e.g., PM_2.5_, CO), it is a technique that can easily be deployed by adding further questions to suit any HAP exposure setting within a questionnaire beyond that of cooking/heating fuel types and location.

The health outcome composite score indicates that exposed children at 7-months may have multiple comorbidities as a result of HAP. No associations were observed at 13 or 36 months with the combined health score, however, due to the differing composition of the score these results cannot be directly compared to those at the 7-month time points. There is little evidence from the literature about multiple comorbidities in children due to exposure to HAP to compare the composite health score to and therefore any composite health score should be interpreted with caution^53^ and validated with further research. Having multiple health conditions associated with exposure to HAP, provides strong evidence for the need to reduce early childhood exposure and policymakers should investigate and consider the wider cost-benefits of HAP reduction in the context of multi-comorbidities.

Undertaking this largescale study on healthy children (i.e., not pre-term, no congenital abnormalities, not low birthweight etc.) may be seen as a limitation to its generalisability in that it excludes vulnerable predispositions such as very pre-mature or very-low birthweight or those with congenital malformations, but has reduced the uncertainties around predispose to events and that healthy children are being affect by HAP exposure. It is anticipated that there is potentially an increased risk of health events with HAP if children are already pre-disposed from birth and therefore our findings are an underestimate of effect that HAP would have at population level; showing the imperative need to develop action against HAP in Mongolia or similar settings to Ulaanbaatar in early 2000s. Further strengths of the study are demonstrated through the use of the WHO ICMI criteria and a large follow-up time. This long follow-up time, however, comes with the risk that household cooking and heating practices could have changed since their assessment at 7-months after birth, and may not be accurate at 36-months.

Furthermore, the composite health measure only indicates that multiple problems can occur as a results of HAP exposure, which provide useful evidence for policy makers. Finally, a strength is that due to all the births occurring within a four-month window (very end of autumn and whole of winter), the impact of season on the health events within this sample are likely to be minimal, apart from pneumonia, as events occurred within the 7-months post-birth. Further exploration would be required to investigate any potential differences if children were born within a different season, as in Ulaanbaatar the summers are warm and for three months there is little need for heating and the gers are more ventilated; even though cooking still takes place indoors with the same stoves.

## Conclusion

Exposure to HAP within this birth cohort in Ulaanbaatar, Mongolia was associated with negative health impacts for child health occurring from birth up to 36-months. Given the proportion of children and women exposed to HAP in Mongolia and the economic and social costs associated with growth retardation and respiratory diseases in children, these results could have significant public health implications and highlights the importance of measure to mitigate HAP exposure in all Mongolian populations and similar Central Asian countries with parallel housing setting. Further work should explore possible mechanisms for the longitudinal association of HAP and child development. In addition, if the HAP composite score is to validated, this could be an accessible method to assess these associations in other LMICs and globally, where indoor combustion of biomass for household energy is still common.

## Methods

### Study area, population and sampling strategy

Data was collected as part of a three-stage RCT in Ulaanbaatar, Mongolia in 2002, which was a large population-based study designed to investigate the effect of swaddling on child development in infants in Mongolia. Detailed documentation of the recruitment and follow-up are described elsewhere^54–56^, but in summary healthy infants delivered in four maternity hospitals in Ulaanbaatar, that manage over 95% of all births in the city, were recruited within 48 h of birth. Infants were excluded if born with a gestation < 36 weeks, a birthweight < 2500 g, severe congenital abnormalities needing special neonatal care, needing intensive care or resided in apartments that were kept too warm for the infant to be swaddled during the daytime. Ethical approval for the study was provided by Ministry of Health of Mongolia and the ethics committee of the London School of Hygiene and Tropical Medicine and fully informed consent was obtained from the mothers. All research was performed in accordance with relevant guidelines/regulations.

**Fig. 1:**
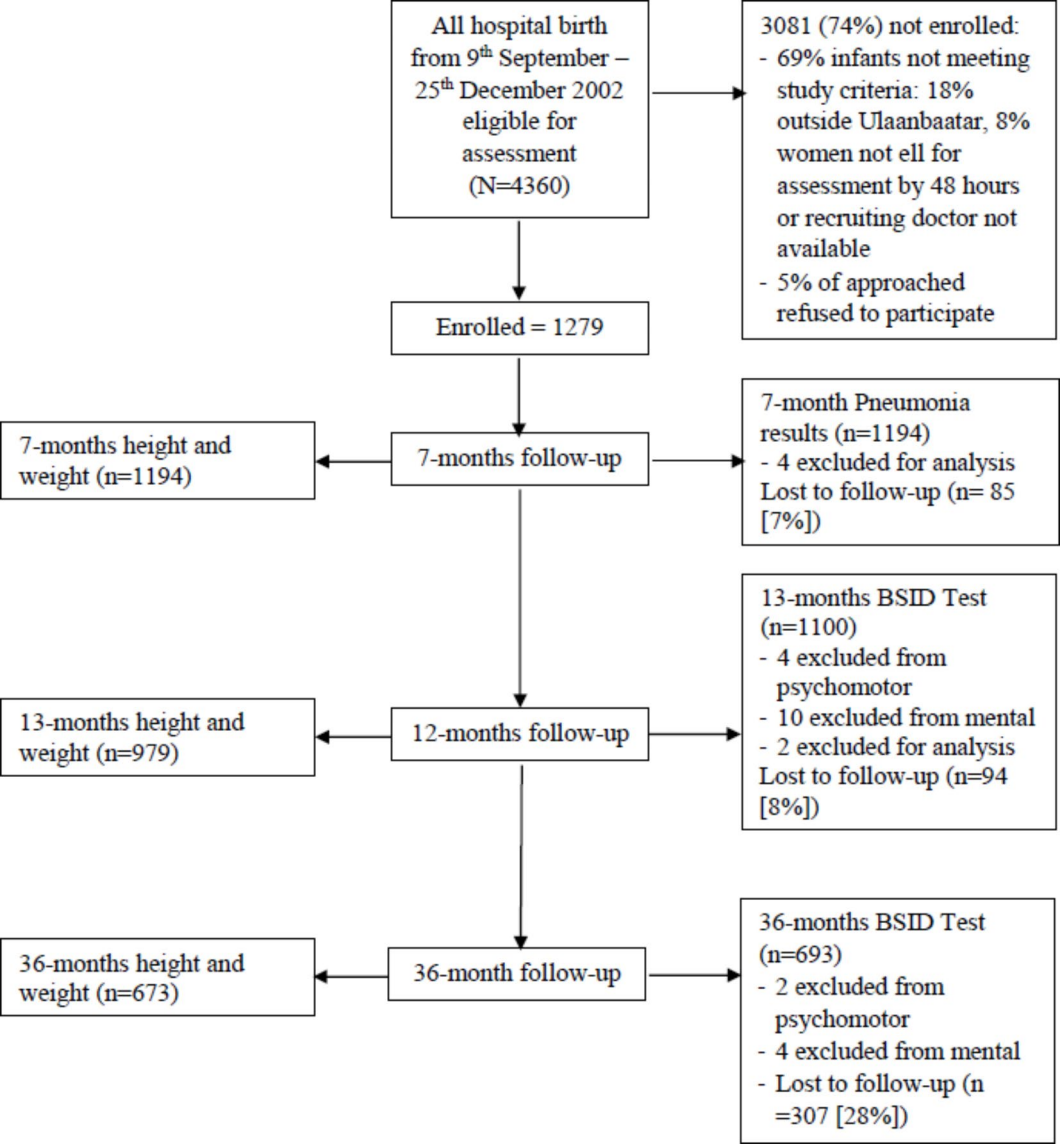
Number of children enrolled, follow-up and outcomes obtained across the 36-month trial period.

Out of 4360 eligible births, 1279 infants were enrolled (Fig. 1), with a total of 1194 infants with pneumonia results at 7-months and BSID data being collected from 1100 infants at 13 months and 693 at 36 months. Except at 36 months, participants were lost to follow up due to refusal to participate, could not be found or had died. At 36 months, due to limited resources, the follow-up was all the families with post-natal depression, plus approximately three times a random sample of the remainder of the families.

### Households air pollution exposure

In a ger household many factors influence the level of HAP present, therefore a weighted composition pollution score (Table 6) was created based on published literature on the effects of pollutant sources^45–47^ (e.g., heating source, cooking fuel, heating fuel, lighting fuel, fuel condition^48,49^) and behaviour^57–59^ (e.g., hours of stove use in 24 h, overnight use of stove, fuel handing practice, length time in 24 h that windows are open^13,48,50,51^) on HAP levels; where the higher the score the greater the level of HAP. Data on household cooking and heating practices were obtained at the 7-month follow-up, via a questionnaire. A corresponding score (Table 6) was given to each characteristic and multiple by the weight, to generate the weighted sum scores^60^. The weights were applied to each HAP factor, with pollutant determinants receiving a larger weighting (i.e., 2) than the behaviour determinants (i.e., 1) as these were perceived to be greater predictors of exposure. Missing values were estimated by taking the average of the total score of each respective variable in accordance with the mean substitution approach^61,62^. As apartments fully rely on heating provided by a central source rather than biomass stoves and cooking was not undertaken on biomass stoves, all apartments were assigned a HAP score of 0. The continuous composite HAP score was further categorised into four levels: lowest (score = 0), low (score < 18), medium (score 18–20), and high (score > 20).

**Table 6:** Scoring system to a create composite HAP exposure score (Max. Total score = 38).

HAP factor	Characteristic	Score	Weight	Max. score
Pollutant determinants (Max. score = 26)^45–47^				
Main heating source	Furnace/internal stove	2	2	4
	External stove	1		
	Electric heater	0		
	Other	1		
	Missing	1		
Fuel used for cooking	Wood	3	2	6
	Coal	2		
	Electricity	0		
	Other	1		
	Missing	1.2		
Fuel used for heating	Wood	3	2	6
	Coal	2		
	Electricity	0		
	Other	1		
	Missing	1.2		
Fuel used for lighting	Wood	3	2	6
	Coal	2		
	Electricity	0		
	Other	1		
	Missing	1.2		
Fuel condition before use^48,49^	Dry/very dry	2	2	4
	Damp/mixed	1		
	Missing	1		
Behavioural determinants (Max. score = 12)^57–59^				
Number of hours stove is on/24 h	Long (16–24 h)	3	1	3
	Medium (< 16 h)	2		
	Short (< 6 h)	1		
	Missing	1.5		
Stove kept alight at night	Yes	3	1	3
	Sometimes	2		
	No	1		
	Missing	1.5		
Practice of handling fuel	Never dry fuel	3	1	3
	Occasionally	2		
	Always	1		
	Missing	1.5		
Practice of handling fuel	Never dry fuel	3	1	3
	Occasionally	2		
	Always	1		
	Missing	1.5		
No. of hours window in room with stove open/24 h^13,48,50,51^	Short (< 3 h)	3	1	3
	Medium (3–6 h)	2		
	Long (> 6 h)	1		
	Missing	1.5		

### Child health outcomes

#### Bayley scales of infant development scores (BSID-II)

The Bayley scales of infant development scores (BSID-II), a widely used and validated tool, was translated into Mongolian (including back-translation, piloting, and expert validation) and extensive training was provided to four child development health specialist professionals who conducted the tests. The BSID-II mental score included: children’s discriminations and response, sensory/perceptual acuity, vocalisation, and verbal communication initiation, memory learning and problem solving, foundations of abstract thinking, object constancy acquisition, complex language, mental mapping, mathematical concepts, and habit formation. Muscle coordination, degree of body control, dynamic movement, postural mimicry, fine manipulation abilities of the hands and fingers, and stereognosis (the ability to recognise objects by touch) are all assessed on the BSID-II psychomotor scale. Both scores are based on the infants reported age with a normed mean of 100 (SD: 15 points). For test-retest reliability, both scales show good correlation coefficients (0.83 and 0.77) for mental and psychomotor assessments^63^.

#### Pneumonia

Childhood pneumonia in children aged 0–7 months was detected through passive and active surveillance. Passive surveillance occurred through primary healthcare centres and hospitals, facilitated using child study ID cards and allocation of a family doctor linked to the study to attend when the child was unwell or the main children’s hospital in an emergency. Transport costs and antibiotics were provided by study as these are not part of the free service provision. Active surveillance occurred during follow-up home-visits through mother’s questionnaires obtaining information on any illnesses since the last visit, their nature and visits to doctor/hospital.

The Integrated Management of Childhood Illness (IMCI) pneumonia definition was used, as it is compared to other studies^64–66^ and is more sensitive (less specific) that other definitions. All trial physicians were re-trained in the IMCI definitions for the purpose of the study. Pulse oximetry and a chest x-ray (undertaken by WHO-trained study radiographers) were undertaken on infants with signs of ‘very severe’ and severe’ pneumonia as differing by the IMCI criteria^64–66^.

#### Height and weight z-scores

Height (cm) and weight (kg) were obtained by trained fieldworkers at 7-, 13-, and 36-months to calculate height-for-age (HAZ) and weight-for-age (WAZ) z-scores based on the WHO classification^67^.

#### Composite health outcome measure

An exploratory composite health score based on the available health outcomes at each of the three time points (7-, 13-, and 36-months) was created to give an indication of the occurrence of multiple health problems associated with HAP exposure. An additional composite score (Table 7) was created based on the occurrence of disease for categorical outcomes and quartiles for continues outcomes. The greater the score the more health events the child experienced, with a maximum score of 10 for 7-months and a maximum score of 16 for both 13- and 36-month timepoints.

**Table 7:** Baseline characteristics categorised by composite household air pollution score.

Health outcome	Included at		Categories	Score
	7-months	13- and 36-month		
Pneumonia	Yes	No	Absent	0
			Occurred once	1
			Multiple occurrences	2
BSID-II mental	No	Yes	1st quartile	4
			2nd quartile	3
			3rd quartile	2
			4th quartile	1
BSID-II psychomotor	No	Yes	1st quartile	4
			2nd quartile	3
			3rd quartile	2
			4th quartile	1
Stunting	Yes	Yes	1st quartile	4
			2nd quartile	3
			3rd quartile	2
			4th quartile	1
Underweight	Yes	Yes	1st quartile	4
			2nd quartile	3
			3rd quartile	2
			4th quartile	1

### Covariates

Child, maternal and environmental confounding factors were considered for analysis, which were all modelled as categorical variables; apart form date of birth which was modelled as a continuous variable. Details of the confounders can be found in Table 1.

### Statistical methods

All data cleaning was undertaken in Microsoft Excel^68^ and analysis in STATA version 17^69^. Descriptive statistic included number of observation (n) and percentage (%) for categorical variables, mean and standard deviation (SD) for normally distributed categorical variables and median and interquartile range (IQR) for skewed continuous variables. To assess the association between HAP and child health outcomes (BSID, pneumonia, HAZ, WAZ, composite health measure) multivariate generalised linear models were used. Covariates were a priori identified, with a hierarchical approach taken to build up the model, starting with child factors, followed by maternal factors and then environmental factors.

## Electronic supplementary material

Below is the link to the electronic supplementary material.

Supplementary Material 1

Supplementary Material 2

## Data Availability

The data that support the findings of this study are available from the corresponding author upon reasonable request.
