# Supplementary material for: Assessing the association between household air pollution exposure and child heath in Mongolia: a birth-cohort study
Source: Sci Rep. 2025 Jan 31;15:3878. doi: 10.1038/s41598-024-79927-6 (PMC11785753; doi:10.1038/s41598-024-79927-6)
Supplement: Supplementary file 1 — Supplementary Material 1 [file 41598_2024_79927_MOESM1_ESM.docx]

| **Table 2.1 Multiple linear regression of the association between HAP scores and Mental scores at 13-months** | | | | | | | | |
| --- | --- | --- | --- | --- | --- | --- | --- | --- |
|  | **Model 1** | |  | **Model 2** | |  | **Model 3** | |
|  | *P* | β (95%CI) |  | *P* | β (95%CI) |  | *P* | β (95%CI) |
| **IAP score** | **0.002** | -0.10 (-0.17, -0.04) |  | **0.049** | -0.08 (-0.16, 0.00) |  | **0.035** | -0.09 (-0.17, -0.01) |
| **Sex (ref = female)** | | | | | | | | |
| Male | 0.388 | 0.57 (-0.72, 1.85) |  | 0.245 | 0.84 (-0.58, 2.26) |  | 0.276 | 0.79 (-0.63, 2.22) |
| **Birthweight (ref = 2400~2950g)** | | | | | | | | |
| 3000~3450g | **0.009** | **2.85 (0.70, 4.99)** |  | **0.005** | **3.46 (1.03, 5.89)** |  | **0.007** | **3.35 (0.90, 5.79)** |
| 3500~3950g | **0.001** | **3.65 (1.44, 5.86)** |  | **0.002** | **4.02 (1.49, 6.55)** |  | **0.002** | **3.96 (1.40, 6.51)** |
| >=4000g | **0.001** | **4.39 (1.69, 7.09)** |  | **0.005** | **4.48 (1.37, 7.58)** |  | **0.006** | **4.39 (1.28, 7.51)** |
| **Gestational age (ref = <38 weeks)** | | | | | | | | |
| >=38weeks | **0.006** | **5.37 (1.53, 9.21)** |  | **0.021** | **4.80 (0.73, 8.87)** |  | **0.02** | **4.83 (0.76, 8.90)** |
| **Date of birth** | **<0.001** | **0.05 (0.03, 0.07)** |  | **<0.001** | **0.05 (0.03, 0.08)** |  | **<0.001** | **0.05 (0.03, 0.08)** |
| **Parity (ref = 0)** | |  |  |  |  |  |  |  |
| 1 or 2 |  |  |  | 0.968 | 0.04 (-2.01, 2.09) |  | 0.853 | 0.19 (-1.87, 2.26) |
| 3 |  |  |  | 0.384 | 0.82 (-1.02, 2.65) |  | 0.446 | 0.72 (-1.13, 2.57) |
| **Maternal marital status (ref = common law)** | | | | | | | | |
| Married |  |  |  | 0.955 | 0.04 (-1.50, 1.59) |  | 0.885 | 0.12 (-1.45, 1.69) |
| Single |  |  |  | 0.778 | -0.70 (-5.61, 4.20) |  | 0.731 | -0.86 (-5.78, 4.06) |
| **Maternal age (ref = <20 years)** | | | | | | | | |
| 20-29 years |  |  |  | 0.39 | -1.30 (-4.27, 1.67) |  | 0.414 | -1.25 (-4.26, 1.76) |
| >=35 years |  |  |  | 0.126 | -2.64 (-6.03, 0.75) |  | 0.13 | -2.66 (-6.10, 0.79) |
| **Maternal education (ref = primary school)** | | | | | | | | |
| Secondary school |  |  |  | 0.084 | 2.14 (-0.29, 4.57) |  | 0.101 | 2.04 (-0.40, 4.48) |
| Tertiary school |  |  |  | 0.089 | 2.32 (-0.36, 4.99) |  | 0.098 | 2.27 (-0.42, 4.97) |
| **Mothers’ employment (ref = No)** | | | | | | | | |
| Yes |  |  |  | 0.942 | 0.07 (-1.78, 1.92) |  | 0.961 | 0.05 (-1.81, 1.90) |
| **Post-natal Depression (ref = Normal)** | | | | | | | | |
| Depressed |  |  |  | 0.263 | -1.12 (-3.10, 0.85) |  | 0.29 | -1.07 (-3.06, 0.92) |
| **Swaddling (ref = Yes)** | | | | | | | | |
| No |  |  |  |  |  |  | 0.282 | -0.78 (-2.19, 0.64) |
| **Type of delivery (ref = Vaginal)** | | | | | | | | |
| Caesarean |  |  |  |  |  |  | 0.352 | -0.91 (-2.82, 1.00) |
| **Breastfeeding until 4-months (ref = Not breastfed)** | | | | | | | | |
| Nonexclusively breastfed |  |  |  |  |  |  | 0.277 | -1.47 (-4.14, 1.19) |
| Exclusively breastfed |  |  |  |  |  |  | 0.325 | -3.50 (-10.46, 3.47) |
| **Number of smokers in household (ref = None)** | | | | | | | | |
| One smoker |  |  |  |  |  |  | 0.286 | -0.83 (-2.37, 0.70) |
| More than one smoker |  |  |  |  |  |  | 0.597 | -0.68 (-3.20, 1.84) |

Footnote:

Model 1: Adjusted for sex, birthweight, gestational age, date of birth.

Model 2: Model 1 plus parity, maternal marital status, maternal age, maternal education, maternal employment, postnatal depression.

Model 3: Model 2 plus type of delivery, swaddling, breastfeeding, number of smokers.

Abbreviations: β = Coefficient, 95% CI = 95% confidence intervals, ref = reference

| **Table 2.2 Multiple linear regression of the association between HAP scores and Psychomotor scores at 13-months** | | | | | | | | |
| --- | --- | --- | --- | --- | --- | --- | --- | --- |
|  | **Model 1** | |  | **Model 2** | |  | **Model 3** | |
|  | *P* | β (95%CI) |  | *P* | β (95%CI) |  | *P* | β (95%CI) |
| **IAP score** | **0.021** | -0.12 (-0.22, -0.02) |  | 0.217 | -0.08 (-0.20, 0.04) |  | 0.154 | -0.09 (-0.21, 0.03) |
| **Sex (ref = female)** | | |  |  |  |  |  |  |
| Male | 0.273 | 1.08 (-0.85, 3.00) |  | 0.241 | 1.28 (-0.86, 3.42) |  | 0.325 | 1.08 (-1.07, 3.22) |
| **Birthweight (ref = 2400~2950g)** | | | | | | | | |
| 3000~3450g | **0.013** | **4.07 (0.85, 7.28)** |  | **0.013** | **4.65 (0.98, 8.32)** |  | **0.02** | **4.37 (0.69, 8.04)** |
| 3500~3950g | **0.003** | **4.99 (1.68, 8.31)** |  | **0.014** | **4.78 (0.95, 8.60)** |  | **0.015** | **4.8 (0.95, 8.64)** |
| >=4000g | **0.016** | **4.96 (0.91, 9.00)** |  | **0.034** | **5.06 (0.37, 9.74)** |  | **0.031** | **5.14 (0.46, 9.82)** |
| **Gestational age (ref = <38 weeks)** | | | | | | | | |
| >=38weeks | **0.026** | **6.45 (0.78, 12.13)** |  | **0.044** | **6.21 (0.17, 12.25)** |  | **0.038** | **6.39 (0.37, 12.42)** |
| **Date of birth** | **0.001** | **0.05 (0.02, 0.08)** |  | **0.001** | **0.06 (0.02, 0.09)** |  | **0.001** | **0.06 (0.03, 0.10)** |
| **Parity (ref = 0)** | | | | | | | | |
| 1 or 2 |  |  |  | 0.345 | -1.48 (-4.56, 1.59) |  | 0.367 | -1.42 (-4.50, 1.67) |
| 3 |  |  |  | 0.257 | -1.60 (-4.36, 1.17) |  | 0.209 | -1.78 (-4.55, 1.00) |
| **Maternal marital status (ref = common law)** | | | | | | | | |
| Married |  |  |  | 0.407 | -0.99 (-3.32, 1.35) |  | 0.539 | -0.74 (-3.09, 1.62) |
| Single |  |  |  | 0.718 | -1.36 (-8.78, 6.05) |  | 0.813 | -0.89 (-8.31, 6.52) |
| **Maternal age (ref = <20 years)** | | | | | | | | |
| 20-29 years |  |  |  | 0.879 | 0.35 (-4.14, 4.83) |  | 0.856 | 0.42 (-4.12, 4.96) |
| >=35 years |  |  |  | 0.656 | -1.16 (-6.28, 3.95) |  | 0.797 | -0.68 (-5.88, 4.52) |
| **Maternal education (ref = primary school)** | | | | | | | | |
| Secondary school |  |  |  | 0.051 | 3.64 (-0.02, 7.29) |  | **0.043** | **3.77 (0.11, 7.43)** |
| Tertiary school |  |  |  | 0.148 | 2.96 (-1.05, 6.97) |  | 0.121 | 3.19 (-0.84, 7.23) |
| **Mothers’ employment (ref = No)** | | | | | | | | |
| Yes |  |  |  | 0.297 | 1.49 (-1.31, 4.28) |  | 0.282 | 1.53 (-1.26, 4.33) |
| **Post-natal Depression (ref = Normal)** | | | | | | | | |
| Depressed |  |  |  | 0.114 | -2.39 (-5.36, 0.57) |  | 0.132 | -2.29 (-5.27, 0.69) |
| **Swaddling (ref = Yes)** | | | | | | | | |
| No |  |  |  |  |  |  | 0.089 | -1.84 (-3.97, 0.28) |
| **Type of delivery (ref = Vaginal)** | | | | | | | | |
| Caesarean |  |  |  |  |  |  | **0.003** | **-4.30 (-7.17, -1.44)** |
| **Breastfeeding until 4-months (ref = Not breastfed)** | | | | | | | | |
| Nonexclusively breastfed |  |  |  |  |  |  | 0.504 | -1.37 (-5.38, 2.64) |
| Exclusively breastfed |  |  |  |  |  |  | 0.988 | 0.08 (-10.42, 10.58) |
| **Number of smokers in household (ref = None)** | | | | | | | | |
| One smoker |  |  |  |  |  |  | 0.935 | -0.10 (-2.40, 2.21) |
| More than one smoker |  |  |  |  |  |  | 0.271 | -2.11 (-5.88, 1.65) |

Footnote:

Model 1: Adjusted for sex, birthweight, gestational age, date of birth.

Model 2: Model 1 plus parity, maternal marital status, maternal age, maternal education, maternal employment, postnatal depression.

Model 3: Model 2 plus type of delivery, swaddling, breastfeeding, number of smokers.

Abbreviations: β = Coefficient, 95% CI = 95% confidence intervals, ref = reference

| **Table 2.3 Multiple linear regression of the association between HAP scores and Mental scores at 36-months** | | | | | | | | |
| --- | --- | --- | --- | --- | --- | --- | --- | --- |
|  | **Model 1** | |  | **Model 2** | |  | **Model 3** | |
|  | *P* | β (95%CI) |  | *P* | β (95%CI) |  | *P* | β (95%CI) |
| **IAP score** | **<0.001** | -0.23 (-0.31, -0.14) |  | 0.115 | -0.08 (-0.18, 0.02) |  | 0.091 | -0.09 (-0.19, 0.01) |
| **Sex (ref = female)** | | | | | | | | |
| Male | **0.043** | 1.72 (0.06, 3.38) |  | 0.054 | 1.77 (-0.03, 3.58) |  | **0.046** | 1.85 (0.04, 3.67) |
| **Birthweight (ref = 2400~2950g)** | | | | | | | | |
| 3000~3450g | **0.019** | **3.43 (0.56, 6.29)** |  | 0.44 | 1.27 (-1.96, 4.49) |  | 0.465 | 1.21 (-2.04, 4.46) |
| 3500~3950g | **0.018** | **3.55 (0.62, 6.48)** |  | 0.499 | 1.13 (-2.15, 4.41) |  | 0.598 | 0.89 (-2.43, 4.21) |
| >=4000g | **0.041** | **3.78 (0.15, 7.41)** |  | 0.81 | 0.51 (-3.65, 4.67) |  | 0.814 | 0.50 (-3.68, 4.68) |
| **Gestational age (ref = <38 weeks)** | | | | | | | | |
| >=38weeks | **0.049** | 4.96 (0.02, 9.89) |  | 0.06 | 4.74 (-0.20, 9.69) |  | 0.057 | 4.82 (-0.15, 9.78) |
| **Date of birth** | 0.521 | -0.01 (-0.04, 0.02) |  | 0.663 | -0.01 (-0.04, 0.02) |  | 0.686 | -0.01 (-0.04, 0.03) |
| **Parity (ref = 0)** | | | | | | | | |
| 1 or 2 |  |  |  | 0.356 | -1.22 (-3.81, 1.37) |  | 0.349 | -1.25 (-3.87, 1.37) |
| 3 |  |  |  | **0.008** | **-3.13 (-5.44, -0.82)** |  | **0.006** | **-3.30 (-5.64, -0.96)** |
| **Maternal marital status (ref = common law)** | | | | | | | | |
| Married |  |  |  | 0.266 | -1.10 (-3.03, 0.84) |  | 0.337 | -0.96 (-2.93, 1.01) |
| Single |  |  |  | 0.09 | -5.07 (-10.93, 0.79) |  | 0.084 | -5.22 (-11.14, 0.70) |
| **Maternal age (ref = <20 years)** | | | | | | | | |
| 20-29 years |  |  |  | 0.427 | -1.53 (-5.33, 2.26) |  | 0.447 | -1.50 (-5.35, 2.36) |
| >=35 years |  |  |  | 0.867 | -0.37 (-4.67, 3.94) |  | 0.847 | -0.44 (-4.85, 3.98) |
| **Maternal education (ref = primary school)** | | | | | | | | |
| Secondary school |  |  |  | **0.001** | **4.97 (1.92, 8.02)** |  | **0.002** | **4.91 (1.83, 7.99)** |
| Tertiary school |  |  |  | **<0.001** | **9.43 (6.10, 12.76)** |  | **<0.001** | **9.36 (5.98, 12.73)** |
| **Mothers’ employment (ref = No)** | | | | | | | | |
| Yes |  |  |  | 0.255 | 1.35 (-0.98, 3.69) |  | 0.296 | 1.25 (-1.10, 3.60) |
| **Post-natal Depression (ref = Normal)** | | | | | | | | |
| Depressed |  |  |  | **0.037** | **-2.83 (-5.48, -0.18)** |  | **0.049** | **-2.70 (-5.39, -0.01)** |
| **Swaddling (ref = Yes)** | | | | | | | | |
| No |  |  |  |  |  |  | 0.565 | -0.53 (-2.36, 1.29) |
| **Type of delivery (ref = Vaginal)** | | | | | | | | |
| Caesarean |  |  |  |  |  |  | 0.693 | 0.51 (-2.02, 3.04) |
| **Breastfeeding until 4-months (ref = Not breastfed)** | | | | | | | | |
| Nonexclusively breastfed |  |  |  |  |  |  | 0.579 | -1.01 (-4.59, 2.57) |
| Exclusively breastfed |  |  |  |  |  |  | 0.764 | -1.53 (-11.51, 8.46) |
| **Number of smokers in household (ref = None)** | | | | | | | | |
| One smoker |  |  |  |  |  |  | 0.812 | 0.23 (-1.68, 2.14) |
| More than one smoker |  |  |  |  |  |  | 0.695 | -0.72 (-4.30, 2.87) |

Footnote:

Model 1: Adjusted for sex, birthweight, gestational age, date of birth.

Model 2: Model 1 plus parity, maternal marital status, maternal age, maternal education, maternal employment, postnatal depression.

Model 3: Model 2 plus type of delivery, swaddling, breastfeeding, number of smokers.

Abbreviations: β = Coefficient, 95% CI = 95% confidence intervals, ref = reference

| **Table 2.4 Multiple linear regression of the association between HAP scores and Psychomotor scores at 36-months** | | | | | | | | |
| --- | --- | --- | --- | --- | --- | --- | --- | --- |
|  | **Model 1** | |  | **Model 2** | |  | **Model 3** | |
|  | *P* | β (95%CI) |  | *P* | β (95%CI) |  | *P* | β (95%CI) |
| **IAP score** | **<0.001** | -0.24 (-0.33, -0.15) |  | **0.011** | -0.13 (-0.23, -0.03) |  | **0.016** | -0.13 (-0.23, -0.02) |
| **Sex (ref = female)** | | | | | | | | |
| Male | 0.653 | -0.39 (-2.08, 1.30) |  | 0.8 | 0.23 (-1.56, 2.02) |  | 0.78 | 0.26 (-1.54, 2.05) |
| **Birthweight (ref = 2400~2950g)** | | | | | | | | |
| 3000~3450g | **0.002** | **4.60 (1.68, 7.51)** |  | **0.021** | **3.78 (0.58, 6.97)** |  | **0.015** | **3.99 (0.78, 7.20)** |
| 3500~3950g | **<0.001** | **5.64 (2.65, 8.63)** |  | **0.002** | **5.05 (1.79, 8.32)** |  | **0.003** | **5.08 (1.79, 8.37)** |
| >=4000g | **0.016** | **4.54 (0.85, 8.23)** |  | 0.126 | 3.21 (-0.90, 7.33) |  | 0.088 | 3.59 (-0.53, 7.71) |
| **Gestational age (ref = <38 weeks)** | | | | | | | | |
| >=38weeks | 0.269 | 2.84 (-2.20, 7.87) |  | 0.335 | 2.41 (-2.50, 7.31) |  | 0.326 | 2.46 (-2.45, 7.37) |
| **Date of birth** | 0.086 | -0.03 (-0.06, 0.00) |  | 0.193 | -0.02 (-0.05, 0.01) |  | 0.272 | -0.02 (-0.05, 0.01) |
| **Parity (ref = 0)** | | | | | | | | |
| 1 or 2 |  |  |  | 0.503 | -0.87 (-3.44, 1.69) |  | 0.424 | -1.05 (-3.63, 1.53) |
| 3 |  |  |  | **0.025** | **-2.61 (-4.90, -0.32)** |  | **0.015** | **-2.87 (-5.18, -0.56)** |
| **Maternal marital status (ref = common law)** | | | | | | | | |
| Married |  |  |  | **0.025** | **-2.19 (-4.10, -0.28)** |  | **0.015** | **-2.42 (-4.36, -0.48)** |
| Single |  |  |  | **0.002** | **-9.20 (-15.02, -3.39)** |  | **0.002** | **-9.31 (-15.16, -3.46)** |
| **Maternal age (ref = <20 years)** | | | | | | | | |
| 20-29 years |  |  |  | 0.092 | -3.23 (-6.99, 0.53) |  | 0.102 | -3.17 (-6.99, 0.64) |
| >=35 years |  |  |  | 0.585 | -1.19 (-5.45, 3.07) |  | 0.676 | -0.93 (-5.29, 3.43) |
| **Maternal education (ref = primary school)** | | | | | | | | |
| Secondary school |  |  |  | **0.029** | **3.37 (0.34, 6.40)** |  | **0.028** | **3.41 (0.36, 6.46)** |
| Tertiary school |  |  |  | **<0.001** | **6.50 (3.19, 9.81)** |  | **<0.001** | **6.32 (2.97, 9.66)** |
| **Mothers’ employment (ref = No)** | | | | | | | | |
| Yes |  |  |  | 0.603 | -0.61 (-2.93, 1.70) |  | 0.584 | -0.65 (-2.97, 1.67) |
| **Post-natal Depression (ref = Normal)** | | | | | | | | |
| Depressed |  |  |  | 0.201 | -1.69 (-4.29, 0.90) |  | 0.179 | -1.80 (-4.43, 0.83) |
| **Swaddling (ref = Yes)** | | | | | | | | |
| No |  |  |  |  |  |  | 0.866 | -0.15 (-1.95, 1.64) |
| **Type of delivery (ref = Vaginal)** | | | | | | | | |
| Caesarean |  |  |  |  |  |  | 0.791 | 0.34 (-2.15, 2.83) |
| **Breastfeeding until 4-months (ref = Not breastfed)** | | | | | | | | |
| Nonexclusively breastfed |  |  |  |  |  |  | 0.165 | 2.47 (-1.02, 5.96) |
| Exclusively breastfed |  |  |  |  |  |  | 0.249 | -5.79 (-15.63, 4.06) |
| **Number of smokers in household (ref = None)** | | | | | | | | |
| One smoker |  |  |  |  |  |  | 0.903 | -0.12 (-2.00, 1.76) |
| More than one smoker |  |  |  |  |  |  | 0.116 | 2.84 (-0.70, 6.39) |

Footnote:

Model 1: Adjusted for sex, birthweight, gestational age, date of birth.

Model 2: Model 1 plus parity, maternal marital status, maternal age, maternal education, maternal employment, postnatal depression.

Model 3: Model 2 plus type of delivery, swaddling, breastfeeding, number of smokers.

Abbreviations: β = Coefficient, 95% CI = 95% confidence intervals, ref = reference

| **Table 3 Survival analysis of the association between HAP and pneumonia** | | | | | | | | |
| --- | --- | --- | --- | --- | --- | --- | --- | --- |
|  | **Model 1** | |  | **Model 2** | |  | **Model 3** | |
|  | *P* | β (95%CI) |  | *P* | β (95%CI) |  | *P* | β (95%CI) |
| **IAP score** | **<0.001** | 1.03 (1.01,1.04) |  | **0.004** | 1.02 (1.01, 1.04) |  | **0.002** | 1.02 (1.01, 1.04) |
| **IAP scale** |  |  |  |  |  |  |  |  |
| Low | **0.036** | **1.43 (1.02, 2.00)** |  | 0.073 | 1.42 (0.97, 2.08) |  | **0.032** | **1.52 (1.04, 2.24)** |
| Medium | **0.001** | **1.82 (1.29, 2.56)** |  | **0.011** | **1.67 (1.13, 2.48)** |  | **0.008** | **1.71 (1.15, 2.55)** |
| High | **<0.001** | **1.91 (1.40, 2.60)** |  | **0.002** | **1.75 (1.22, 2.52)** |  | **0.002** | **1.80 (1.25, 2.59)** |
| **Sex (ref = female)** | | | | | | | | |
| Male | **0.008** | **0.73 (0.58, 0.92)** |  | **0.01** | **0.72 (0.56, 0.92)** |  | **0.012** | **0.72 (0.56, 0.93)** |
| **Birthweight (ref = 2400~2950g)** | | | | | | | | |
| 3000~3450g | 0.058 | 0.71 (0.50, 1.01) |  | 0.083 | 0.71 (0.48, 1.05) |  | 0.176 | 0.76 (0.51, 1.13) |
| 3500~3950g | **0.035** | **0.68 (0.47, 0.97)** |  | 0.144 | 0.74 (0.49, 1.11) |  | 0.186 | 0.75 (0.5, 1.15) |
| >=4000g | 0.26 | 0.77 (0.49, 1.21) |  | 0.63 | 0.88 (0.53, 1.46) |  | 0.725 | 0.91 (0.55, 1.52) |
| **Gestational age (ref = <38 weeks)** | | | | | | | | |
| >=38weeks | 0.676 | 1.15 (0.59, 2.25) |  | 0.462 | 1.31 (0.64, 2.67) |  | 0.504 | 1.28 (0.62, 2.61) |
| **Date of birth** | 0.419 | 1.00 (1.00, 1.01) |  | 0.718 | 1.00 (1.00, 1.01) |  | 0.756 | 1.00 (1.00, 1.01) |
| **Parity (ref = 0)** |  |  |  |  |  |  |  |  |
| 1 or 2 |  |  |  | 0.405 | 1.16 (0.82, 1.63) |  | 0.573 | 1.10 (0.78, 1.56) |
| 3 |  |  |  | 0.636 | 1.08 (0.78, 1.49) |  | 0.693 | 1.07 (0.77, 1.48) |
| **Maternal marital status (ref = common law)** | | | | | | | | |
| Married |  |  |  | 0.509 | 1.10 (0.84, 1.43) |  | 0.504 | 1.10 (0.83, 1.44) |
| Single |  |  |  | 0.718 | 0.86 (0.37, 1.99) |  | 0.842 | 0.92 (0.39, 2.15) |
| **Maternal age (ref = <20 years)** | | | | | | | | |
| 20-29 years |  |  |  | 0.88 | 0.96 (0.61, 1.54) |  | 0.698 | 1.10 (0.69, 1.76) |
| >=35 years |  |  |  | 0.1 | 0.62 (0.35, 1.10) |  | 0.252 | 0.71 (0.4, 1.27) |
| **Maternal education (ref = primary school)** | | | | | | | | |
| Secondary school |  |  |  | 0.155 | 0.77 (0.53, 1.11) |  | 0.196 | 0.79 (0.54, 1.13) |
| Tertiary school |  |  |  | **0.044** | **0.64 (0.42, 0.99)** |  | 0.051 | 0.65 (0.42, 1.00) |
| **Mothers’ employment (ref = No)** | | | | | | | | |
| Yes |  |  |  | 0.09 | 0.77 (0.57, 1.04) |  | 0.075 | 0.76 (0.56, 1.03) |
| **Post-natal Depression (ref = Normal)** | | | | | | | | |
| Depressed |  |  |  | **0.001** | **1.68 (1.24, 2.28)** |  | **0.002** | **1.62 (1.19, 2.21)** |
| **Swaddling (ref = Yes)** | | | | | | | | |
| No |  |  |  |  |  |  | **0.024** | **1.34 (1.04, 1.73)** |
| **Type of delivery (ref = Vaginal)** | | | | | | | | |
| Caesarean |  |  |  |  |  |  | 0.214 | 0.79 (0.54, 1.15) |
| **Breastfeeding until 4-months (ref = Not breastfed)** | | | | | | | | |
| Nonexclusively breastfed |  |  |  |  |  |  | 0.283 | 0.77 (0.47, 1.25) |
| Exclusively breastfed |  |  |  |  |  |  | **0.007** | **3.39 (1.39, 8.28)** |
| **Number of smokers in household (ref = None)** | | | | | | | | |
| One smoker |  |  |  |  |  |  | 0.101 | 1.26 (0.96, 1.67) |
| More than one smoker |  |  |  |  |  |  | **0.025** | **1.57 (1.06, 2.34)** |

Footnote:

Model 1: Adjusted for sex, birthweight, gestational age, date of birth.

Model 2: Model 1 plus parity, maternal marital status, maternal age, maternal education, maternal employment, postnatal depression.

Model 3: Model 2 plus type of delivery, swaddling, breastfeeding, number of smokers.

Abbreviations: β = Coefficient, 95% CI = 95% confidence intervals, ref = reference

| **Table 4.1 Multiple linear regression of the association between HAP scores with height for age z score (HAZ) at 7-months** | | | | | | | | |
| --- | --- | --- | --- | --- | --- | --- | --- | --- |
|  | **Model 1** | |  | **Model 2** | |  | **Model 3** | |
|  | *P* | β (95%CI) |  | *P* | β (95%CI) |  | *P* | β (95%CI) |
| **IAP score** | **<0.001** | **-0.03 (-0.04, -0.02)** |  | **<0.001** | **-0.02 (-0.03, -0.01)** |  | **<0.001** | **-0.02 (-0.03, -0.01)** |
| **Sex (ref = female)** | |  |  |  |  |  |  |  |
| Males | **<0.001** | **0.32 (0.16, 0.48)** |  | **0.001** | **0.29 (0.12, 0.46)** |  | **0.001** | **0.28 (0.11, 0.46)** |
| **Birthweight (ref = 2400~2950g)** | | | | | | | | |
| 3000~3450g | **<0.001** | **0.64 (0.37, 0.92)** |  | **<0.001** | **0.62 (0.311, 0.92)** |  | **<0.001** | **0.58 (0.28, 0.89)** |
| 3500~3950g | **<0.001** | **1.04 (0.76, 1.32)** |  | **<0.001** | **1.08 (0.77, 1.39)** |  | **<0.001** | **1.04 (0.73, 1.36)** |
| >=4000g | **<0.001** | **1.63 (1.30, 1.97)** |  | **<0.001** | **1.61 (1.23, 2.00)** |  | **<0.001** | **1.62 (1.23, 2.00)** |
| **Gestational age (ref = <38 weeks)** | | |  |  |  |  |  |  |
| >=38weeks | 0.297 | 0.24 (-0.21, 0.69) |  | 0.144 | 0.34 (-0.12, 0.79) |  | 0.15 | 0.33 (-0.12, 0.79) |
| **Date of birth** | **0.029** | **0.00 (-0.01, 0.00)** |  | **0.028** | **0.00 (-0.01, 0.00)** |  | **0.024** | **0.00 (-0.01, 0.00)** |
| **Parity (ref = 0)** |  |  |  |  |  |  |  |  |
| 1 or 2 |  |  |  | 0.345 | -0.12 (-0.36, 0.13) |  | 0.36 | -0.11 (-0.36, 0.13) |
| 3 |  |  |  | **0.001** | **-0.38 (-0.60, -0.16)** |  | **0.002** | **-0.36 (-0.59, -0.14)** |
| **Maternal marital status (ref = common law)** | | | | |  |  |  |  |
| Married |  |  |  | 0.564 | -0.05 (-0.24, 0.13) |  | 0.938 | -0.01 (-0.20, 0.18) |
| Single |  |  |  | 0.359 | -0.26 (-0.81, 0.29) |  | 0.356 | -0.26 (-0.82, 0.29) |
| **Maternal age (ref = <20 years)** | | |  |  |  |  |  |  |
| 20-29 years |  |  |  | 0.118 | -0.30 (-0.67, 0.08) |  | 0.057 | -0.37 (-0.75, 0.01) |
| >=35 years |  |  |  | 0.135 | -0.32 (-0.74, 0.10) |  | 0.081 | -0.38 (-0.81, 0.05) |
| **Maternal education (ref = primary school)** | | |  |  |  |  |  |  |
| Secondary school |  |  |  | **<0.001** | **0.54 (0.25, 0.82)** |  | **<0.001** | **0.53 (0.24, 0.81)** |
| Tertiary school |  |  |  | **<0.001** | **0.64 (0.33, 0.96)** |  | **<0.001** | **0.61 (0.29, 0.93)** |
| **Mothers’ employment (ref = No)** | | |  |  |  |  |  |  |
| Yes |  |  |  | 0.611 | 0.06 (-0.16, 0.27) |  | 0.579 | 0.06 (-0.16, 0.28) |
| **Post-natal Depression (ref = Normal)** | | |  |  |  |  |  |  |
| Depressed |  |  |  | 0.481 | -0.09 (-0.33, 0.16) |  | 0.692 | -0.05 (-0.30, 0.20) |
| **Swaddling (ref = Yes)** | | |  |  |  |  |  |  |
| No |  |  |  | **0.03** | **58.64 (5.74, 111.54)** |  | 0.69 | -0.03 (-0.21, 0.14) |
| **Type of delivery (ref = Vaginal)** | | |  |  |  |  |  |  |
| Caesarean |  |  |  |  |  |  | 0.967 | -0.01 (-0.25, 0.24) |
| **Breastfeeding until 4-months (ref = Not breastfed)** | | | | |  |  |  |  |
| Nonexclusively breastfed |  |  |  |  |  |  | 0.301 | 0.18 (-0.16, 0.52) |
| Exclusively breastfed |  |  |  |  |  |  | 0.93 | 0.07 (-1.56, 1.71) |
| **Number of smokers in household (ref = None)** | | | | |  |  |  |  |
| One smoker |  |  |  |  |  |  | 0.061 | -0.18 (-0.37, 0.01) |
| More than one smoker |  |  |  |  |  |  | **0.039** | **-0.32 (-0.62, -0.02)** |

Footnote:

Model 1: Adjusted for sex, birthweight, gestational age, date of birth.

Model 2: Model 1 plus parity, maternal marital status, maternal age, maternal education, maternal employment, postnatal depression.

Model 3: Model 2 plus type of delivery, swaddling, breastfeeding, number of smokers.

Abbreviations: β = Coefficient, 95% CI = 95% confidence intervals, ref = reference

| **Table 4.2 Multiple linear regression of the association between HAP scores with weight for age z-score (WAZ) at 7-months** | | | | | | | | |
| --- | --- | --- | --- | --- | --- | --- | --- | --- |
|  | **Model 1** | |  | **Model 2** | |  | **Model 3** | |
|  | *P* | β (95%CI) |  | *P* | β (95%CI) |  | *P* | β (95%CI) |
| **IAP score** | **<0.001** | **-0.01 (-0.02, -0.01)** |  | **0.04** | **-0.01 (-0.02, 0.00)** |  | 0.059 | -0.01 (-0.02, 0.00) |
| **Sex (ref = female)** | | |  |  |  |  |  |  |
| Male | **0.019** | **0.16 (0.03, 0.29)** |  | 0.209 | 0.09 (-0.05, 0.24) |  | 0.259 | 0.08 (-0.06, 0.23) |
| **Birthweight (ref = 2400~2950g)** | | |  |  |  |  |  |  |
| 3000~3450g | **<0.001** | **0.57 (0.34, 0.80)** |  | **<0.001** | **0.51 (0.25, 0.76)** |  | **<0.001** | **0.49 (0.24, 0.75)** |
| 3500~3950g | **<0.001** | **1.02 (0.78, 1.25)** |  | **<0.001** | **0.93 (0.66, 1.20)** |  | **<0.001** | **0.91 (0.64, 1.18)** |
| >=4000g | **<0.001** | **1.42 (1.14, 1.71)** |  | **<0.001** | **1.34 (1.01, 1.67)** |  | **<0.001** | **1.35 (1.02, 1.68)** |
| **Gestational age (ref = <38 weeks)** | | |  |  |  |  |  |  |
| >=38weeks | 0.75 | -0.06 (-0.44, 0.31) |  | 0.995 | 0.00 (-0.38, 0.39) |  | 0.941 | -0.01 (-0.4, 0.37) |
| **Date of birth** | 0.108 | 0.00 (0.00, 0.00) |  | 0.052 | 0.00 (-0.01, 0.00) |  | **0.043** | **0.00 (-0.01, 0.00)** |
| **Parity (ref = 0)** |  |  |  |  |  |  |  |  |
| 1 or 2 |  |  |  | **0.035** | **-0.22 (-0.43, -0.02)** |  | 0.057 | -0.20 (-0.41, 0.01) |
| 3 |  |  |  | 0.075 | -0.17 (-0.36, 0.02) |  | 0.142 | -0.14 (-0.33, 0.05) |
| **Maternal marital status (ref = common law)** | | | | |  |  |  |  |
| Married |  |  |  | 0.72 | -0.03 (-0.19, 0.13) |  | 0.873 | -0.01 (-0.17, 0.15) |
| Single |  |  |  | 0.623 | 0.12 (-0.36, 0.60) |  | 0.74 | 0.08 (-0.4, 0.56) |
| **Maternal age (ref = <20 years)** | | |  |  |  |  |  |  |
| 20-29 years |  |  |  | 0.603 | 0.08 (-0.24, 0.41) |  | 0.996 | 0.00 (-0.32, 0.32) |
| >=35 years |  |  |  | 0.675 | 0.08 (-0.28, 0.44) |  | 0.883 | -0.03 (-0.39, 0.34) |
| **Maternal education (ref = primary school)** | | | | |  |  |  |  |
| Secondary school |  |  |  | **0.007** | **0.33 (0.09, 0.57)** |  | **0.008** | **0.33 (0.09, 0.57)** |
| Tertiary school |  |  |  | **0.01** | **0.36 (0.09, 0.63)** |  | **0.015** | **0.34 (0.06, 0.61)** |
| **Mothers’ employment (ref = No)** | | | |  |  |  |  |  |
| Yes |  |  |  | 0.586 | 0.05 (-0.13, 0.23) |  | 0.48 | 0.07 (-0.12, 0.25) |
| **Post-natal Depression (ref = Normal)** | | |  |  |  |  |  |  |
| Depressed |  |  |  | 0.052 | -0.20 (-0.41, 0.00) |  | 0.078 | -0.19 (-0.39, 0.02) |
| **Swaddling (ref = Yes)** | | |  |  |  |  |  |  |
| No |  |  |  | 0.053 | 44.15 (-0.61, 88.91) |  | 0.263 | 0.08 (-0.06, 0.23) |
| **Type of delivery (ref = Vaginal)** | | | |  |  |  |  |  |
| Caesarean |  |  |  |  |  |  | 0.141 | 0.16 (-0.05, 0.36) |
| **Breastfeeding until 4-months (ref = Not breastfed)** | | | | |  |  |  |  |
| Nonexclusively breastfed |  |  |  |  |  |  | **0.009** | **0.38 (0.09, 0.67)** |
| Exclusively breastfed |  |  |  |  |  |  | 0.506 | 0.47 (-0.92, 1.86) |
| **Number of smokers in household (ref = None)** | | | | | |  |  |  |
| One smoker |  |  |  |  |  |  | 0.104 | -0.13 (-0.29, 0.03) |
| More than one smoker |  |  |  |  |  |  | 0.074 | -0.23 (-0.49, 0.02) |

Footnote:

Model 1: Adjusted for sex, birthweight, gestational age, date of birth.

Model 2: Model 1 plus parity, maternal marital status, maternal age, maternal education, maternal employment, postnatal depression.

Model 3: Model 2 plus type of delivery, swaddling, breastfeeding, number of smokers.

Abbreviations: β = Coefficient, 95% CI = 95% confidence intervals, ref = reference

| **Table 4.3 Multiple linear regression of the association between HAP scores with height for age z score (HAZ) at 13-months** | | | | | | | | |
| --- | --- | --- | --- | --- | --- | --- | --- | --- |
|  | **Model 1** | |  | **Model 2** | |  | **Model 3** | |
|  | *P* | β (95%CI) |  | *P* | β (95%CI) |  | *P* | β (95%CI) |
| **IAP score** | **<0.001** | **-0.03 (-0.03, -0.02)** |  | **<0.001** | **-0.02 (-0.03, -0.01)** |  | **<0.001** | **-0.02 (-0.03, -0.01)** |
| **Sex (ref = female)** | |  |  |  |  |  |  |  |
| Male | **<0.001** | **0.30 (0.16, 0.44)** |  | **<0.001** | **0.32 (0.16, 0.48)** |  | **<0.001** | **0.30 (0.14, 0.46)** |
| **Birthweight (ref = 2400~2950g)** | | |  |  |  |  |  |  |
| 3000~3450g | **<0.001** | **0.48 (0.25, 0.71)** |  | **0.003** | **0.41 (0.14, 0.69)** |  | **0.004** | **0.40 (0.13, 0.67)** |
| 3500~3950g | **<0.001** | **0.80 (0.56, 1.03)** |  | **<0.001** | **0.69 (0.41, 0.97)** |  | **<0.001** | **0.67 (0.39, 0.96)** |
| >=4000g | **<0.001** | **1.22 (0.93, 1.52)** |  | **<0.001** | **1.10 (0.75, 1.46)** |  | **<0.001** | **1.11 (0.76, 1.47)** |
| **Gestational age (ref = <38 weeks)** | | |  |  |  |  |  |  |
| >=38weeks | 0.416 | 0.17 (-0.24, 0.59) |  | 0.855 | 0.04 (-0.41, 0.50) |  | 0.874 | 0.04 (-0.42, 0.49) |
| **Date of birth** | 0.245 | 0.00 (0.00, 0.00) |  | 0.297 | 0.00 (0.00, 0.00) |  | 0.333 | 0.00 (0.00, 0.00) |
| **Parity (ref = 0)** |  |  |  |  |  |  |  |  |
| 1 or 2 |  |  |  | 0.226 | -0.14 (-0.37, 0.09) |  | 0.264 | -0.13 (-0.36, 0.10) |
| 3 |  |  |  | 0.089 | -0.18 (-0.38, 0.03) |  | 0.133 | -0.16 (-0.37, 0.05) |
| **Maternal marital status (ref = common law)** | | | | |  |  |  |  |
| Married |  |  |  | 0.806 | 0.02 (-0.15, 0.2) |  | 0.677 | 0.04 (-0.14, 0.22) |
| Single |  |  |  | 0.603 | 0.14 (-0.40, 0.69) |  | 0.613 | 0.14 (-0.41, 0.69) |
| **Maternal age (ref = <20 years)** | | |  |  |  |  |  |  |
| 20-29 years |  |  |  | 0.735 | -0.06 (-0.40, 0.28) |  | 0.377 | -0.16 (-0.50, 0.19) |
| >=35 years |  |  |  | 0.91 | -0.02 (-0.41, 0.36) |  | 0.487 | -0.14 (-0.54, 0.25) |
| **Maternal education (ref = primary school)** | | |  |  |  |  |  |  |
| Secondary school |  |  |  | **0.013** | **0.34 (0.07, 0.61)** |  | **0.017** | **0.33 (0.06, 0.6)** |
| Tertiary school |  |  |  | **0.001** | **0.49 (0.19, 0.78)** |  | **0.004** | **0.44 (0.15, 0.74)** |
| **Mothers’ employment (ref = No)** | | |  |  |  |  |  |  |
| Yes |  |  |  | 0.28 | 0.12 (-0.09, 0.33) |  | 0.281 | 0.12 (-0.10, 0.33) |
| **Post-natal Depression (ref = Normal)** | | |  |  |  |  |  |  |
| Depressed |  |  |  | 0.06 | -0.21 (-0.44, 0.01) |  | 0.142 | -0.17 (-0.40, 0.06) |
| **Swaddling (ref = Yes)** | | |  |  |  |  |  |  |
| No |  |  |  | 0.329 | 19.93 (-20.15, 60.02) |  | 0.575 | -0.05 (-0.21, 0.11) |
| **Type of delivery (ref = Vaginal)** | | |  |  |  |  |  |  |
| Caesarean |  |  |  |  |  |  | 0.206 | 0.14 (-0.08, 0.35) |
| **Breastfeeding until 4-months (ref = Not breastfed)** | | | | | |  |  |  |
| Nonexclusively breastfed |  |  |  |  |  |  | 0.414 | 0.13 (-0.18, 0.43) |
| Exclusively breastfed |  |  |  |  |  |  | 0.392 | -0.35 (-1.14, 0.45) |
| **Number of smokers in household (ref = None)** | | | | | |  |  |  |
| One smoker |  |  |  |  |  |  | 0.179 | -0.12 (-0.29, 0.05) |
| More than one smoker |  |  |  |  |  |  | **0.034** | **-0.31 (-0.59, -0.02)** |

Footnote:

Model 1: Adjusted for sex, birthweight, gestational age, date of birth.

Model 2: Model 1 plus parity, maternal marital status, maternal age, maternal education, maternal employment, postnatal depression.

Model 3: Model 2 plus type of delivery, swaddling, breastfeeding, number of smokers.

Abbreviations: β = Coefficient, 95% CI = 95% confidence intervals, ref = reference

| **Table 4.4 Multiple linear regression of the association between HAP scores with weight for age z-score (WAZ) at 13-months** | | | | | | | | |
| --- | --- | --- | --- | --- | --- | --- | --- | --- |
|  | **Model 1** | |  | **Model 2** | |  | **Model 3** | |
|  | *P* | β (95%CI) |  | *P* | β (95%CI) |  | *P* | β (95%CI) |
| **IAP score** | **<0.001** | **-0.01 (-0.02, -0.01)** |  | **0.002** | **-0.01 (-0.02, 0.00)** |  | **0.002** | **-0.01 (-0.02, 0.00)** |
| **Sex (ref = female)** | | |  |  |  |  |  |  |
| Male | 0.07 | 0.11 (-0.01, 0.23) |  | 0.112 | 0.11 (-0.03, 0.24) |  | 0.169 | 0.09 (-0.04, 0.22) |
| **Birthweight (ref = 2400~2950g)** | | |  |  |  |  |  |  |
| 3000~3450g | **<0.001** | **0.42 (0.22, 0.61)** |  | **0.002** | **0.36 (0.14, 0.58)** |  | **0.001** | **0.37 (0.15, 0.59)** |
| 3500~3950g | **<0.001** | **0.76 (0.56, 0.96)** |  | **<0.001** | **0.68 (0.45, 0.91)** |  | **<0.001** | **0.69 (0.46, 0.93)** |
| >=4000g | **<0.001** | **1.11 (0.86, 1.36)** |  | **<0.001** | **1.03 (0.74, 1.33)** |  | **<0.001** | **1.05 (0.76, 1.34)** |
| **Gestational age (ref = <38 weeks)** | | |  |  |  |  |  |  |
| >=38weeks | 0.63 | 0.08 (-0.26, 0.43) |  | 0.974 | -0.01 (-0.38, 0.37) |  | 0.957 | -0.01 (-0.38, 0.36) |
| **Date of birth** | 0.07 | 0.00 (0.00, 0.00) |  | 0.105 | 0.00 (0.00, 0.00) |  | 0.098 | 0.00 (0.00, 0.00) |
| **Parity (ref = 0)** |  |  |  |  |  |  |  |  |
| 1 or 2 |  |  |  | 0.891 | -0.01 (-0.20, 0.17) |  | 0.979 | 0.00 (-0.19, 0.19) |
| 3 |  |  |  | 0.537 | -0.05 (-0.22, 0.12) |  | 0.724 | -0.03 (-0.20, 0.14) |
| **Maternal marital status (ref = common law)** | | | | |  |  |  |  |
| Married |  |  |  | 0.801 | 0.02 (-0.13, 0.16) |  | 0.766 | 0.02 (-0.12, 0.17) |
| Single |  |  |  | 0.283 | 0.24 (-0.20, 0.69) |  | 0.266 | 0.25 (-0.19, 0.70) |
| **Maternal age (ref = <20 years)** | | | |  |  |  |  |  |
| 20-29 years |  |  |  | 0.741 | 0.05 (-0.23, 0.33) |  | 0.855 | -0.03 (-0.31, 0.26) |
| >=35 years |  |  |  | 0.774 | -0.05 (-0.36, 0.27) |  | 0.361 | -0.15 (-0.47, 0.17) |
| **Maternal education (ref = primary school)** | | |  |  |  |  |  |  |
| Secondary school |  |  |  | 0.094 | 0.19 (-0.03, 0.41) |  | 0.09 | 0.19 (-0.03, 0.41) |
| Tertiary school |  |  |  | **0.013** | **0.31 (0.06, 0.55)** |  | **0.021** | **0.29 (0.04, 0.53)** |
| **Mothers’ employment (ref = No)** | | |  |  |  |  |  |  |
| Yes |  |  |  | 0.649 | 0.04 (-0.13, 0.21) |  | 0.664 | 0.04 (-0.13, 0.21) |
| **Post-natal Depression (ref = Normal)** | | |  |  |  |  |  |  |
| Depressed |  |  |  | 0.291 | -0.10 (-0.28, 0.08) |  | 0.483 | -0.07 (-0.25, 0.12) |
| **Swaddling (ref = Yes)** | | |  |  |  |  |  |  |
| No |  |  |  | 0.109 | 26.87 (-6.05, 59.78) |  | 0.492 | 0.05 (-0.08, 0.18) |
| **Type of delivery (ref = Vaginal)** | | |  |  |  |  |  |  |
| Caesarean |  |  |  |  |  |  | 0.138 | 0.13 (-0.04, 0.31) |
| **Breastfeeding until 4-months (ref = Not breastfed)** | | | | |  |  |  |  |
| Nonexclusively breastfed |  |  |  |  |  |  | 0.545 | 0.08 (-0.17, 0.33) |
| Exclusively breastfed |  |  |  |  |  |  | 0.414 | -0.27 (-0.92, 0.38) |
| **Number of smokers in household (ref = None)** | | | | |  |  |  |  |
| One smoker |  |  |  |  |  |  | 0.89 | -0.01 (-0.15, 0.13) |
| More than one smoker |  |  |  |  |  |  | **0.016** | **-0.28 (-0.52, -0.05)** |

Footnote:

Model 1: Adjusted for sex, birthweight, gestational age, date of birth.

Model 2: Model 1 plus parity, maternal marital status, maternal age, maternal education, maternal employment, postnatal depression.

Model 3: Model 2 plus type of delivery, swaddling, breastfeeding, number of smokers.

Abbreviations: β = Coefficient, 95% CI = 95% confidence intervals, ref = reference

| **Table 4.5 Multiple linear regression of the association between HAP scores with height for age z score (HAZ) at 36-months** | | | | | | | | |
| --- | --- | --- | --- | --- | --- | --- | --- | --- |
|  | **Model 1** | |  | **Model 2** | |  | **Model 3** | |
|  | *P* | β (95%CI) |  | *P* | β (95%CI) |  | *P* | β (95%CI) |
| **IAP score** | **<0.001** | **-0.02 (-0.03, -0.01)** |  | **0.015** | **-0.01 (-0.02, 0.00)** |  | **0.021** | **-0.01 (-0.02, 0.00)** |
| **Sex (ref = female)** | | |  |  |  |  |  |  |
| Male | **<0.001** | **-0.45 (-0.59, -0.30)** |  | **<0.001** | **-0.39 (-0.55, -0.23)** |  | **<0.001** | **-0.4 (-0.56, -0.24)** |
| **Birthweight (ref = 2400~2950g)** | | |  |  |  |  |  |  |
| 3000~3450g | **0.001** | **0.43 (0.18, 0.68)** |  | **0.012** | **0.36 (0.08, 0.64)** |  | **0.015** | **0.35 (0.07, 0.63)** |
| 3500~3950g | **<0.001** | **0.63 (0.37, 0.88)** |  | **<0.001** | **0.57 (0.28, 0.85)** |  | **<0.001** | **0.55 (0.26, 0.84)** |
| >=4000g | **<0.001** | **0.96 (0.63, 1.28)** |  | **<0.001** | **0.81 (0.45, 1.18)** |  | **<0.001** | **0.84 (0.47, 1.21)** |
| **Gestational age (ref = <38 weeks)** | | |  |  |  |  |  |  |
| >=38weeks | 0.889 | -0.03 (-0.48, 0.41) |  | 0.949 | 0.01 (-0.43, 0.46) |  | 0.913 | 0.02 (-0.42, 0.47) |
| **Date of birth** | 0.883 | 0.00 (0.00, 0.00) |  | 0.744 | 0.00 (0.00, 0.00) |  | 0.59 | 0.00 (0.00, 0.00) |
| **Parity (ref = 0)** |  |  |  |  |  |  |  |  |
| 1 or 2 |  |  |  | 0.542 | 0.07 (-0.16, 0.30) |  | 0.625 | 0.06 (-0.17, 0.29) |
| 3 |  |  |  | **0.018** | **-0.25 (-0.45, -0.04)** |  | **0.022** | **-0.24 (-0.45, -0.03)** |
| **Maternal marital status (ref = common law)** | | | | |  |  |  |  |
| Married |  |  |  | 0.157 | -0.12 (-0.29, 0.05) |  | 0.156 | -0.13 (-0.30, 0.05) |
| Single |  |  |  | 0.897 | 0.03 (-0.49, 0.56) |  | 0.929 | 0.02 (-0.51, 0.56) |
| **Maternal age (ref = <20 years)** | | | | |  |  |  |  |
| 20-29 years |  |  |  | **0.019** | **-0.40 (-0.74, -0.07)** |  | **0.008** | **-0.46 (-0.8, -0.12)** |
| >=35 years |  |  |  | 0.121 | -0.30 (-0.68, 0.08) |  | 0.06 | -0.37 (-0.76, 0.02) |
| **Maternal education (ref = primary school)** | | | | | |  |  |  |
| Secondary school |  |  |  | **<0.001** | **0.56 (0.3, 0.83)** |  | **<0.001** | **0.56 (0.29, 0.83)** |
| Tertiary school |  |  |  | **<0.001** | **0.79 (0.5, 1.08)** |  | **<0.001** | **0.76 (0.46, 1.05)** |
| **Mothers’ employment (ref = No)** | | |  |  |  |  |  |  |
| Yes |  |  |  | 0.806 | -0.03 (-0.23, 0.18) |  | 0.759 | -0.03 (-0.24, 0.17) |
| **Post-natal Depression (ref = Normal)** | | |  |  |  |  |  |  |
| Depressed |  |  |  | 0.07 | -0.21 (-0.43, 0.02) |  | 0.114 | -0.18 (-0.41, 0.04) |
| **Swaddling (ref = Yes)** | | |  |  |  |  |  |  |
| No |  |  |  | 0.709 | -8.20 (-51.27, 34.87) |  | 0.587 | -0.04 (-0.20, 0.12) |
| **Type of delivery (ref = Vaginal)** | | |  |  |  |  |  |  |
| Caesarean |  |  |  |  |  |  | 0.283 | 0.12 (-0.10, 0.35) |
| **Breastfeeding until 4-months (ref = Not breastfed)** | | | | |  |  |  |  |
| Nonexclusively breastfed |  |  |  |  |  |  | 0.232 | 0.19 (-0.12, 0.51) |
| Exclusively breastfed |  |  |  |  |  |  | 0.266 | -0.51 (-1.40, 0.39) |
| **Number of smokers in household (ref = None)** | | | | | |  |  |  |
| One smoker |  |  |  |  |  |  | 0.406 | -0.07 (-0.24, 0.10) |
| More than one smoker |  |  |  |  |  |  | 0.412 | -0.13 (-0.45, 0.18) |

Footnote:

Model 1: Adjusted for sex, birthweight, gestational age, date of birth.

Model 2: Model 1 plus parity, maternal marital status, maternal age, maternal education, maternal employment, postnatal depression.

Model 3: Model 2 plus type of delivery, swaddling, breastfeeding, number of smokers.

Abbreviations: β = Coefficient, 95% CI = 95% confidence intervals, ref = reference

| **Table 4.6 Multiple linear regression of the association between HAP scores with weight for age z-score (WAZ) at 36-months** | | | | | | | | |
| --- | --- | --- | --- | --- | --- | --- | --- | --- |
|  | **Model 1** | |  | **Model 2** | |  | **Model 3** | |
|  | *P* | β (95%CI) |  | *P* | β (95%CI) |  | *P* | β (95%CI) |
| **IAP score** | **<0.001** | **-0.01 (-0.02, -0.01)** |  | **0.043** | **-0.01 (-0.01, 0.00)** |  | 0.052 | -0.01 (-0.01, 0.00) |
| **Sex (ref = female)** | | |  |  |  |  |  |  |
| Male | **<0.001** | **-0.41 (-0.53, -0.30)** |  | **<0.001** | **-0.42 (-0.54, -0.29)** |  | **<0.001** | **-0.43 (-0.56, -0.30)** |
| **Birthweight (ref = 2400~2950g)** | | |  |  |  |  |  |  |
| 3000~3450g | **0.003** | **0.30 (0.10, 0.49)** |  | **0.033** | **0.24 (0.02, 0.46)** |  | **0.035** | **0.24 (0.02, 0.46)** |
| 3500~3950g | **<0.001** | **0.60 (0.40, 0.80)** |  | **<0.001** | **0.57 (0.34, 0.79)** |  | **<0.001** | **0.55 (0.32, 0.78)** |
| >=4000g | **<0.001** | **0.93 (0.69, 1.18)** |  | **<0.001** | **0.92 (0.64, 1.21)** |  | **<0.001** | **0.94 (0.65, 1.23)** |
| **Gestational age (ref = <38 weeks)** | | |  |  |  |  |  |  |
| >=38weeks | 0.811 | 0.04 (-0.30, 0.39) |  | 0.711 | 0.07 (-0.28, 0.42) |  | 0.69 | 0.07 (-0.28, 0.42) |
| **Date of birth** | 0.148 | 0.00 (0.00, 0.00) |  | 0.2 | 0.00 (0.00, 0.00) |  | 0.249 | 0.00 (0.00, 0.00) |
| **Parity (ref = 0)** |  |  |  |  |  |  |  |  |
| 1 or 2 |  |  |  | 0.98 | 0.00 (-0.18, 0.18) |  | 0.966 | 0.00 (-0.19, 0.18) |
| 3 |  |  |  | 0.147 | -0.12 (-0.28, 0.04) |  | 0.179 | -0.11 (-0.28, 0.05) |
| **Maternal marital status (ref = common law)** | | | | |  |  |  |  |
| Married |  |  |  | 0.937 | -0.01 (-0.14, 0.13) |  | 0.82 | -0.02 (-0.15, 0.12) |
| Single |  |  |  | 0.122 | 0.33 (-0.09, 0.74) |  | 0.103 | 0.35 (-0.07, 0.77) |
| **Maternal age (ref = <20 years)** | | |  |  |  |  |  |  |
| 20-29 years |  |  |  | 0.49 | -0.09 (-0.36, 0.17) |  | 0.304 | -0.14 (-0.41, 0.13) |
| >=35 years |  |  |  | 0.323 | -0.15 (-0.45, 0.15) |  | 0.165 | -0.22 (-0.52, 0.09) |
| **Maternal education (ref = primary school)** | | | | |  |  |  |  |
| Secondary school |  |  |  | **0.005** | **0.31 (0.09, 0.52)** |  | **0.005** | **0.30 (0.09, 0.51)** |
| Tertiary school |  |  |  | **0.01** | **0.30 (0.07, 0.53)** |  | **0.027** | **0.26 (0.03, 0.49)** |
| **Mothers’ employment (ref = No)** | | |  |  |  |  |  |  |
| Yes |  |  |  | 0.377 | -0.07 (-0.24, 0.09) |  | 0.408 | -0.07 (-0.23, 0.09) |
| **Post-natal Depression (ref = Normal)** | | | | | |  |  |  |
| Depressed |  |  |  | **0.022** | **-0.20 (-0.38, -0.03)** |  | **0.036** | **-0.19 (-0.37, -0.01)** |
| **Swaddling (ref = Yes)** | | |  |  |  |  |  |  |
| No |  |  |  | 0.202 | 22.07 (-11.89, 56.02) |  | 0.282 | 0.07 (-0.06, 0.20) |
| **Type of delivery (ref = Vaginal)** | | | | | |  |  |  |
| Caesarean |  |  |  |  |  |  | 0.401 | 0.08 (-0.10, 0.25) |
| **Breastfeeding until 4-months (ref = Not breastfed)** | | | | | |  |  |  |
| Nonexclusively breastfed |  |  |  |  |  |  | **0.019** | **0.30 (0.05, 0.55)** |
| Exclusively breastfed |  |  |  |  |  |  | 0.944 | -0.03 (-0.73, 0.68) |
| **Number of smokers in household (ref = None)** | | | | |  |  |  |  |
| One smoker |  |  |  |  |  |  | 0.312 | -0.07 (-0.20, 0.06) |
| More than one smoker |  |  |  |  |  |  | 0.372 | -0.11 (-0.36, 0.14) |

Footnote:

Model 1: Adjusted for sex, birthweight, gestational age, date of birth.

Model 2: Model 1 plus parity, maternal marital status, maternal age, maternal education, maternal employment, postnatal depression.

Model 3: Model 2 plus type of delivery, swaddling, breastfeeding, number of smokers.

Abbreviations: β = Coefficient, 95% CI = 95% confidence intervals, ref = reference

| **Table 5.1 Multiple linear regression of the association between HAP and composite health score at 7-months** | | | | | | | | |
| --- | --- | --- | --- | --- | --- | --- | --- | --- |
|  | **Model 1** | |  | **Model 2** | |  | **Model 3** | |
|  | *P* | β (95%CI) |  | *P* | β (95%CI) |  | *P* | β (95%CI) |
| **IAP score** | **<0.001** | **0.03 (0.02, 0.04)** |  | **0.009** | **0.02 (0.01, 0.04)** |  | **0.013** | **0.02 (0.00, 0.04)** |
| **Sex (ref = female)** |  |  |  |  |  |  |  |  |
| Male | 0.067 | -0.24 (-0.49, 0.02) |  | 0.376 | -0.13 (-0.40, 0.15) |  | 0.45 | -0.11 (-0.39, 0.17) |
| **Birthweight (ref = 2400~2950g)** | | |  |  |  |  |  |  |
| 3000~3450g | **<0.001** | **-1.00 (-1.44, -0.57)** |  | **<0.001** | **-0.91 (-1.40, -0.43)** |  | **<0.001** | **-0.89 (-1.38, -0.40)** |
| 3500~3950g | **<0.001** | **-1.86 (-2.31, -1.41)** |  | **<0.001** | **-1.79 (-2.29, -1.28)** |  | **<0.001** | **-1.75 (-2.27, -1.24)** |
| >=4000g | **<0.001** | **-2.82 (-3.36, -2.27)** |  | **<0.001** | **-2.75 (-3.36, -2.13)** |  | **<0.001** | **-2.77 (-3.39, -2.15)** |
| **Gestational age (ref = <38 weeks)** | | |  |  |  |  |  |  |
| >=38weeks | 0.716 | -0.13 (-0.84, 0.58) |  | 0.392 | -0.32 (-1.04, 0.41) |  | 0.424 | -0.30 (-1.02, 0.43) |
| **Date of birth** | **0.01** | **0.01 (0.00, 0.01)** |  | **0.004** | **0.01 (0.00, 0.01)** |  | **0.004** | **0.01 (0.00, 0.01)** |
| **Parity (ref = 0)** |  |  |  |  |  |  |  |  |
| 1 or 2 |  |  |  | 0.063 | 0.37 (-0.02, 0.76) |  | 0.078 | 0.35 (-0.04, 0.75) |
| 3 |  |  |  | **0.014** | **0.45 (0.09, 0.80)** |  | **0.024** | **0.42 (0.05, 0.78)** |
| **Maternal marital status (ref = common law)** | | | | |  |  |  |  |
| Married |  |  |  | 0.886 | -0.02 (-0.32, 0.28) |  | 0.639 | -0.07 (-0.38, 0.23) |
| Single |  |  |  | 0.638 | -0.22 (-1.14, 0.70) |  | 0.699 | -0.18 (-1.11, 0.75) |
| **Maternal age (ref = <20 years)** | | | | |  |  |  |  |
| 20-29 years |  |  |  | 0.943 | -0.02 (-0.63, 0.58) |  | 0.765 | 0.09 (-0.52, 0.71) |
| >=35 years |  |  |  | 0.642 | 0.16 (-0.52, 0.84) |  | 0.426 | 0.28 (-0.41, 0.97) |
| **Maternal education (ref = primary school)** | | | | |  |  |  |  |
| Secondary school |  |  |  | **0.019** | **-0.54 (-1.00, -0.09)** |  | **0.022** | **-0.54 (-1.00, -0.08)** |
| Tertiary school |  |  |  | **0.035** | **-0.55 (-1.06, -0.04)** |  | 0.057 | -0.50 (-1.01, 0.01) |
| **Mothers’ employment (ref = No)** | | | | |  |  |  |  |
| Yes |  |  |  | 0.577 | -0.10 (-0.45, 0.25) |  | 0.515 | -0.12 (-0.47, 0.23) |
| **Post-natal Depression (ref = Normal)** | | | | |  |  |  |  |
| Depressed |  |  |  | 0.479 | 0.14 (-0.25, 0.53) |  | 0.616 | 0.10 (-0.30, 0.50) |
| **Swaddling (ref = Yes)** | | |  |  |  |  |  |  |
| No |  |  |  |  |  |  | 0.565 | -0.08 (-0.36, 0.20) |
| **Type of delivery (ref = Vaginal)** | | |  |  |  |  |  |  |
| Caesarean |  |  |  |  |  |  | 0.658 | -0.09 (-0.48, 0.31) |
| **Breastfeeding until 4-months (ref = Not breastfed)** | | | | |  |  |  |  |
| Nonexclusively breastfed |  |  |  |  |  |  | 0.089 | -0.47 (-1.01, 0.07) |
| Exclusively breastfed |  |  |  |  |  |  | 0.603 | -0.69 (-3.30, 1.92) |
| **Number of smokers in household (ref = None)** | | | | |  |  |  |  |
| One smoker |  |  |  |  |  |  | 0.166 | 0.21 (-0.09, 0.52) |
| More than one smoker |  |  |  |  |  |  | 0.119 | 0.39 (-0.10, 0.87) |

Footnote:

Model 1: Adjusted for sex, birthweight, gestational age, date of birth.

Model 2: Model 1 plus parity, maternal marital status, maternal age, maternal education, maternal employment, postnatal depression.

Model 3: Model 2 plus type of delivery, swaddling, breastfeeding, number of smokers.

Abbreviations: β = Coefficient, 95% CI = 95% confidence intervals, ref = reference

| **Table 5.2 Multiple linear regression of the association between HAP and composite health score at 13-months** | | | | | | | | |
| --- | --- | --- | --- | --- | --- | --- | --- | --- |
|  | **Model 1** | |  | **Model 2** | |  | **Model 3** | |
|  | *P* | β (95%CI) |  | *P* | β (95%CI) |  | *P* | β (95%CI) |
| **IAP score** | **0.005** | **0.03 (0.01, 0.04)** |  | **0.033** | **0.02 (0.00, 0.04)** |  | 0.056 | 0.02 (0.00, 0.04) |
| **Sex (ref = female)** |  |  |  |  |  |  |  |  |
| Male | 0.251 | -0.20 (-0.55, 0.14) |  | 0.699 | -0.08 (-0.46, 0.31) |  | 0.696 | -0.08 (-0.46, 0.31) |
| **Birthweight (ref = 2400~2950g)** | | |  |  |  |  |  |  |
| 3000~3450g | 0.235 | -0.35 (-0.92, 0.23) |  | 0.736 | -0.11 (-0.76, 0.54) |  | 0.682 | -0.14 (-0.79, 0.52) |
| 3500~3950g | **0.004** | **-0.87 (-1.46, -0.28)** |  | 0.092 | -0.58 (-1.26, 0.10) |  | 0.098 | -0.58 (-1.26, 0.11) |
| >=4000g | **<0.001** | **-1.66 (-2.39, -0.92)** |  | **<0.001** | **-1.47 (-2.31, -0.62)** |  | **0.001** | **-1.46 (-2.31, -0.61)** |
| **Gestational age (ref = <38 weeks)** | | |  |  |  |  |  |  |
| >=38weeks | 0.055 | 1.00 (-0.02, 2.01) |  | 0.07 | 1.00 (-0.08, 2.08) |  | 0.057 | 1.05 (-0.03, 2.12) |
| **Date of birth** | **<0.001** | **0.01 (0.01, 0.02)** |  | **<0.001** | **0.01 (0.005, 0.02)** |  | **<0.001** | **0.01 (0.01, 0.02)** |
| **Parity (ref = 0)** |  |  |  |  |  |  |  |  |
| 1 or 2 |  |  |  | 0.727 | -0.10 (-0.65, 0.45) |  | 0.691 | -0.11 (-0.67, 0.44) |
| 3 |  |  |  | 0.896 | -0.03 (-0.53, 0.46) |  | 0.706 | -0.10 (-0.59, 0.40) |
| **Maternal marital status (ref = common law)** | | |  |  |  |  |  |  |
| Married |  |  |  | 0.259 | -0.24 (-0.66, 0.18) |  | 0.252 | -0.25 (-0.67, 0.18) |
| Single |  |  |  | 0.075 | -1.21 (-2.53, 0.12) |  | 0.082 | -1.18 (-2.51, 0.15) |
| **Maternal age (ref = <20 years)** | | |  |  |  |  |  |  |
| 20-29 years |  |  |  | 0.535 | -0.26 (-1.08, 0.56) |  | 0.619 | -0.21 (-1.04, 0.62) |
| >=35 years |  |  |  | 0.497 | -0.32 (-1.25, 0.61) |  | 0.704 | -0.18 (-1.13, 0.77) |
| **Maternal education (ref = primary school)** | | |  |  |  |  |  |  |
| Secondary school |  |  |  | 0.754 | -0.10 (-0.75, 0.54) |  | 0.783 | -0.09 (-0.74, 0.56) |
| Tertiary school |  |  |  | 0.095 | -0.61 (-1.32, 0.11) |  | 0.132 | -0.55 (-1.27, 0.17) |
| **Mothers’ employment (ref = No)** | | |  |  |  |  |  |  |
| Yes |  |  |  | 0.998 | 0.00 (-0.50, 0.5) |  | 0.902 | 0.03 (-0.47, 0.54) |
| **Post-natal Depression (ref = Normal)** | | |  |  |  |  |  |  |
| Depressed |  |  |  | 0.627 | 0.13 (-0.40, 0.67) |  | 0.723 | 0.10 (-0.44, 0.64) |
| **Swaddling (ref = Yes)** | |  |  |  |  |  |  |  |
| No |  |  |  |  |  |  | 0.184 | -0.26 (-0.64, 0.12) |
| **Type of delivery (ref = Vaginal)** | | |  |  |  |  |  |  |
| Caesarean |  |  |  |  |  |  | **0.017** | **-0.62 (-1.13, -0.11)** |
| **Breastfeeding until 4-months (ref = Not breastfed)** | | | | |  |  |  |  |
| Nonexclusively breastfed |  |  |  |  |  |  | 0.994 | 0.00 (-0.73, 0.73) |
| Exclusively breastfed |  |  |  |  |  |  | 0.493 | 0.65 (-1.22, 2.53) |
| **Number of smokers in household (ref = None)** | | | |  |  |  |  |  |
| One smoker |  |  |  |  |  |  | 0.636 | 0.10 (-0.32, 0.51) |
| More than one smoker |  |  |  |  |  |  | 0.767 | 0.10 (-0.57, 0.78) |

Footnote:

Model 1: Adjusted for sex, birthweight, gestational age, date of birth.

Model 2: Model 1 plus parity, maternal marital status, maternal age, maternal education, maternal employment, postnatal depression.

Model 3: Model 2 plus type of delivery, swaddling, breastfeeding, number of smokers

Abbreviations: β = Coefficient, 95% CI = 95% confidence intervals, ref = reference

| **Table 5.3 Multiple linear regression of the association between HAP and composite health score at 36-months** | | | | | | | | |
| --- | --- | --- | --- | --- | --- | --- | --- | --- |
|  | **Model 1** | |  | **Model 2** | |  | **Model 3** | |
|  | *P* | β (95%CI) |  | *P* | β (95%CI) |  | *P* | β (95%CI) |
| **IAP score** | 0.414 | -0.01 (-0.03, 0.01) |  | 0.996 | 0.00 (-0.02, 0.02) |  | 0.955 | 0.00 (-0.02, 0.02) |
| **Sex (ref = female)** |  |  |  |  |  |  |  |  |
| Male | **<0.001** | **1.18 (0.81, 1.55)** |  | **<0.001** | **1.23 (0.81,21.65)** |  | **<0.001** | **1.28 (0.86, 1.69)** |
| **Birthweight (ref = 2400~2950g)** |  |  |  |  |  |  |  |  |
| 3000~3450g | 0.744 | -0.11 (-0.74, 0.53) |  | 0.58 | -0.21 (-0.95, 0.53) |  | 0.583 | -0.21 (-0.96, 0.54) |
| 3500~3950g | 0.072 | -0.60 (-1.25, 0.05) |  | **0.047** | **-0.77 (-1.53, -0.01)** |  | **0.049** | **-0.77 (-1.53, 0.00)** |
| >=4000g | **0.004** | **-1.20 (-2.01, -0.39)** |  | **0.002** | **-1.54 (-2.50, -0.58)** |  | **0.002** | **-1.55 (-2.51, -0.58)** |
| **Gestational age (ref = <38 weeks)** |  |  |  |  |  |  |  |  |
| >=38weeks | 0.121 | 0.86 (-0.23, 1.94) |  | 0.214 | 0.71 (-0.41, 1.83) |  | 0.223 | 0.70 (-0.43, 1.82) |
| **Date of birth** | 0.754 | 0.00 (-0.01, 0.01) |  | 0.926 | 0.00 (-0.01, 0.01) |  | 0.885 | 0.00 (-0.01, 0.01) |
| **Parity (ref = 0)** |  |  |  |  |  |  |  |  |
| 1 or 2 |  |  |  | 0.672 | -0.13 (-0.72, 0.46) |  | 0.656 | -0.13 (-0.73, 0.46) |
| 3 |  |  |  | 0.645 | -0.12 (-0.66, 0.41) |  | 0.45 | -0.21 (-0.74, 0.33) |
| **Maternal marital status (ref = common law)** | | | | | | | | |
| Married |  |  |  | 0.623 | -0.11 (-0.55, 0.33) |  | 0.749 | -0.07 (-0.52, 0.38) |
| Single |  |  |  | **0.006** | **-1.87 (-3.20, -0.54)** |  | **0.007** | **-1.86 (-3.20, -0.52)** |
| **Maternal age (ref = <20 years)** | | | | | | | | |
| 20-29 years |  |  |  | 0.84 | 0.09 (-0.77, 0.95) |  | 0.658 | 0.20 (-0.68, 1.07) |
| >=35 years |  |  |  | 0.342 | 0.47 (-0.50, 1.45) |  | 0.181 | 0.68 (-0.32, 1.69) |
| **Maternal education (ref = primary school)** | | | | | | | | |
| Secondary school |  |  |  | 0.647 | 0.16 (-0.54, 0.86) |  | 0.698 | 0.14 (-0.56, 0.84) |
| Tertiary school |  |  |  | 0.298 | 0.40 (-0.36, 1.17) |  | 0.258 | 0.44 (-0.33, 1.22) |
| **Mothers’ employment (ref = No)** |  |  |  |  |  |  |  |  |
| Yes |  |  |  | 0.968 | -0.01 (-0.55, 0.52) |  | 0.959 | -0.01 (-0.55, 0.52) |
| **Post-natal Depression (ref = Normal)** | | | | | | | | |
| Depressed |  |  |  | 0.199 | 0.40 (-0.21, 1.00) |  | 0.25 | 0.36 (-0.25, 0.97) |
| **Swaddling (ref = Yes)** | | | | | | | | |
| No |  |  |  |  |  |  | 0.559 | -0.12 (-0.54, 0.29) |
| **Type of delivery (ref = Vaginal)** |  |  |  |  |  |  |  |  |
| Caesarean |  |  |  |  |  |  | 0.25 | -0.34 (-0.91, 0.24) |
| **Breastfeeding until 4-months (ref = Not breastfed)** | | | | | | | | |
| Nonexclusively breastfed |  |  |  |  |  |  | 0.384 | -0.36 (-1.17, 0.45) |
| Exclusively breastfed |  |  |  |  |  |  | 0.847 | 0.22 (-2.04, 2.48) |
| **Number of smokers in household (ref = None)** | | | | | | | | |
| One smoker |  |  |  |  |  |  | 0.902 | 0.03 (-0.41, 0.46) |
| More than one smoker |  |  |  |  |  |  | 0.521 | 0.27 (-0.55, 1.08) |

Footnote:

Model 1: Adjusted for Sex, Birthweight, Gestational age, date of birth

Model 2: Model 1 plus Parity, Maternal marital status, Maternal age, Maternal education, Maternal employment, Postnatal depression

Model 3: Model 2 plus Type of delivery, Swaddling, Breastfeeding, Number of smokers

Abbreviations: β = Coefficient, 95% CI = 95% confidence intervals, ref = reference
