# Supplementary material for: Assessing the association between household air pollution exposure and child heath in Mongolia: a birth-cohort study
Source: Sci Rep. 2025 Jan 31;15:3878. doi: 10.1038/s41598-024-79927-6 (PMC11785753; doi:10.1038/s41598-024-79927-6)
Supplement: Supplementary file 2 — Supplementary Material 2 [file 41598_2024_79927_MOESM2_ESM.docx]

| **Appendix 2: Multiple linear regression of the association between HAP and composite health score at 36-months, with adjustment for residence within the 36-month follow-up period** | | |
| --- | --- | --- |
|  | **Model 1** | |
|  | *P* | β (95%CI) |
| **IAP score** | 0·657 | -0·01 (-0·02, 0·03) |
| **Sex (ref = female)** |  |  |
| Male | **<0·001** | 1·25 (0·83, 1·69) |
| **Birthweight (ref = 2400~2950g)** |  |  |
| 3000~3450g | 0·516 | -0·25 (-0·99, 0·50) |
| 3500~3950g | 0·052 | -0·76 (-1·52, 0·01) |
| >=4000g | **0·003** | -1·49 (-2·46, -0·52) |
| **Gestational age (ref = <38 weeks)** |  |  |
| >=38weeks | 0·236 | 0·68 (-0·45, 1·80) |
| **Date of birth** | 0·819 | -0·00 (-0·01, 0·01) |
| **Parity (ref = 0)** |  |  |
| 1 or 2 | 0.521 | -0.20 (-0.79, 0.40) |
| 3 | 0.481 | -0.19 (-0.73, 0.35) |
| **Maternal marital status (ref = common law)** | | |
| Married | 0.735 | -0.80 (-0.53, 0.38) |
| Single | **0.006** | -1.89 ( -3.23, -0.55) |
|  |  |  |
| **Maternal age (ref = <20 years)** | | |
| 20-29 years | 0.793 | 0.12 ( -0.77, 1.00) |
| >=35 years | 0.289 | 0.55 (-0.47, 1.56) |
| **Maternal education (ref = primary school)** | | |
| Secondary school | 0.664 | 0.16 (-0.55, 0.86) |
| Tertiary school | 0.233 | 0.47 (-0.31, 1.25) |
| **Mothers’ employment (ref = No)** |  |  |
| Yes | 0.931 | -0.24 (-0.56, 0.52) |
| **Post-natal Depression (ref = Normal)** | | |
| Depressed | 0.260 | 0.35 (-0.26, 0.97) |
| **Swaddling (ref = Yes)** | | |
| No | 0.756 | -0.66 ( -0.49, 0.35 |
| **Type of delivery (ref = Vaginal)** |  |  |
| Caesarean | 0.273 | -0.19 (-0.90, 0.26) |
| **Breastfeeding until 4 months (ref = Not breastfed)** | | |
| Nonexclusively breastfed | 0.390 | -0.36 ( -1.17, 0.46) |
| Exclusively breastfed | 0.880 | 0.17 ( -2.08, 2.43) |
| **Number of smokers in household (ref = None)** | | |
| One smoker | 0.995 | -0.01 (-0.45, 0.43) |
| More than one smoker | 0.627 | 0.20 (-0.62, 1.02) |
| **Moved within follow-up period (ref=0)** |  |  |
| Once | 0.325 | 0.48 (-0.44, 1.44) |
| Twice | 0.785 | -0.12 (-0.98, 0.74) |
| Footnote:  Model 1: Adjusted for sex, birthweight, gestational age, date of birth.  Model 2: Model 1 plus parity, maternal marital status, maternal age, maternal education, maternal employment, postnatal depression.  Model 3: Model 2 plus type of delivery, swaddling, breastfeeding, number of smokers, change of residence  Abbreviations: β = Coefficient, 95% CI = 95% confidence intervals, ref = reference | | |
